# Cbl-Associated Protein CAP contributes to correct formation and robust function of the *Drosophila* heart tube

**DOI:** 10.1371/journal.pone.0233719

**Published:** 2020-05-29

**Authors:** Jennifer Jammrath, Ingolf Reim, Harald Saumweber

**Affiliations:** 1 Max Delbrück Center for Molecular Medicine, Berlin, Germany; 2 Division of Cytogenetics, Institute of Biology, Humboldt University Berlin, Berlin, Germany; 3 Division of Developmental Biology, Department of Biology, Friedrich-Alexander University of Erlangen-Nürnberg, Erlangen, Germany; 4 Muscle Research Center Erlangen (MURCE), Erlangen, Germany; Imperial College London, UNITED KINGDOM

## Abstract

The formation of a tube-like structure is a basic step in the making of functional hearts in vertebrates and invertebrates and therefore, its understanding provides important information on heart development and function. In *Drosophila*, the cardiac tube originates from two bilateral rows of dorsally migrating cells. On meeting at the dorsal midline, coordinated changes in cell shape and adhesive properties transform the two sheets of cells into a linear tube. ECM and transmembrane proteins linked to the cytoskeleton play an important role during these dynamic processes. Here we characterize the requirement of Cbl-Associated Protein (CAP) in *Drosophila* heart formation. In embryos, CAP is expressed in late migrating cardioblasts and is located preferentially at their luminal and abluminal periphery. *CAP* mutations result in irregular cardioblast alignment and imprecisely controlled cardioblast numbers. Furthermore, *CAP* mutant embryos show a strongly reduced heart lumen and an aberrant shape of lumen forming cardioblasts. Analysis of double heterozygous animals reveals a genetic interaction of *CAP* with Integrin- and Talin-encoding genes. In post-embryonic stages, CAP closely colocalizes with Integrin near Z-bands and at cell–cell contact sites. *CAP* mutants exhibit a reduced contractility in larval hearts and show a locally disrupted morphology, which correlates with a reduced pumping efficiency. Our observations imply a function of CAP in linking Integrin signaling with the actin cytoskeleton. As a modulator of the cytoskeleton, CAP is involved in the establishment of proper cell shapes during cardioblast alignment and cardiac lumen formation in the *Drosophila* embryo. Furthermore, CAP is required for correct heart function throughout development.

## Introduction

Significant insights in heart development and function at the molecular-genetic level were obtained by study of model organisms. The morphogenetic origin, structure and function of the *Drosophila* heart (dorsal vessel, DV) exhibits remarkable conservation with vertebrates [1–4] and has been instructive in revealing mechanisms of cardiomyopathies and aging [5–8]. The *Drosophila* heart derives from cardiac progenitors located in bilateral anlagen within the dorsal mesoderm. After their specification, cardioblasts (CBs) align as two lateral rows of migrating cells that eventually meet and line up at the dorsal midline ([9]; reviewed by [10–14]). Approaching the dorsal midline, the contralateral CBs polarize by reorganization of their extracellular matrix (ECM), transmembrane receptors and associated intracellular proteins, forming a leading edge and later distinct basal, junctional (J) and luminal (L) domains ([15–18]; see below). Contralateral CBs make first contact at their dorsal, cadherin-rich J domain. After dorsal sealing their cell shape changes from pear-like to crescent-like due to cytoskeletal remodeling [17]. By this event, the ventral sides of contralateral CBs come in close proximity, allowing establishment of cell contacts between their ventral J domains and a heart lumen forms between the non-adherent L domains [9, 19].

The wall of the DV is formed by 52 pairs of CBs, which are flanked by non-contractile, more loosely arranged pericardial cells that have nephrocytic functions. The posterior one-third of the DV, referred to as the heart, is distinguished from the aorta [20] by a wider lumen and by the differentiation of specialized CBs (ostial cells) into inflow tracts for the hemolymph. At its boundary to the aorta a valve, formed by another pair of special cells, inhibits retrograde flow from the aorta, the outflow tract of the heart [9, 10, 13]. Late in embryo development, the cardiomyoblasts organize a contractile apparatus in form of striated circular myofibrils. In the resulting cardiomyocytes (CMCs) these fibrils are embedded and anchored in the dorsal and ventral contact zone of contralateral cells, organized by the Integrin Adhesion Complex (IAC, see below). There, the myofibrils line up and become matched in register with the myofibrils of the contralateral cells [7]. In the larvae, the principal organization of the DV is maintained, but there is an eightfold hypertrophic growth of CMCs with a concomitant increase in the number of contractile myofibrils [7, 21]. During metamorphosis the most posterior cells of the heart undergo apoptosis. The aortal part takes over heart function, becomes widened and four pairs of ostia progenitor CMCs form new inflow tracts. In addition, three new intracardial valves are formed [22, 23]. In addition to the CMCs with a circular arrangement of myofibrils, a subset of heart tube-connected striated muscles (alary muscles) transdifferentiates into a layer of longitudinal myofibers associated with the ventral surface of the heart tube [21, 24].

Recent years have seen a significant progress in establishing molecular mechanisms that contribute to the heart assembly and lumen formation. The polarization of CBs is essential for correct heart tube morphogenesis [15–18]. During migration, the leading edge actively extends filopodia, where non-muscle Myosin II (Zipper), the Rho-GTPase Cdc42 and the formins dDAAM and Diaphanous are located [25]. This domain forms the dorsal J domain, the site of first contact between contralateral CBs. The dorsal and ventral J domains are characterized by the expression of adherens junction proteins β-Catenin (Armadillo) and E-cadherin (Shotgun). The non-adhesive L domain between the J domains displays the ECM protein Laminin and its receptor Integrin [16, 18, 26–28]. Integrin is an anchoring as well as signaling molecule that is required for adhesion and tension signaling to the cytoskeleton through links to the ECM established by the Integrin Adhesion Complex (see below). At this early stage Integrin is required for polarization and recruitment of Robo1/2 and its ligand, the signaling molecule Slit [29–31]. Slit and Robo in turn are essential for the formation of a non-adhesive luminal domain. The J and L domains maintain themselves by negative interactions and provide the cells with local information to regulate the adhesive properties and the cell shape changes required for tube and lumen formation [17, 18]. In addition, the Netrins together with their receptors Unc5 [32] or Frazzled [33] provide cues independent form the Slit/Robo pathway for DV tubulogenesis. Netrin signaling mediated by Frazzled facilitates cell shape changes and attachment of contralateral CBs [33].

Integrins can directly interact with actin, but normally this interaction is supported by adapter proteins like Talin, Vinculin and Paxillin [34–36]. These and other unknown adapter proteins together with Integrin-linked kinases form the Integrin Adhesion Complex (IAC). The IAC links and modulates integrin adhesion and signaling across the cell membrane to the cytoskeleton by regulating the affinity for ligands and recruiting numerous modifying proteins [37]. Talin (encoded by *rhea*) together with Integrin is an early marker of the L domain and supports Integrin in the localization of Slit and Robo [38]. Like Integrin, Talin is continuously required for heart growth to maintain the one to one apposition of myofibril ends between cardiomyocytes. The induced knock down of Integrin or Talin results in degeneration of cardiomyocyte contacts and a reduction in myofibril length and number [6, 7].

We report here the function of CAP in the development of the *Drosophila* DV. *Drosophila* CAP is an orthologue of the conserved mammalian CAP protein, an adapter protein of the CAV family (CAP/Ponsin, ArgBP2 and Vinexin; [39]). The CAV proteins regulate cell adhesion, cytoskeletal organization, and growth factor signaling. They contain a N-terminal SoHo (Sorbin homology) domain that was found to interact with Flotillin in lipid rafts in HEK293T cells in vitro [40]. Their three C-terminal SH3 domains mediate the interaction with multiple binding partners including signaling molecules and cytoskeletal proteins [41–44]. CAP was first described by its association with the c-Cbl protein, an E3 ubiquitin ligase involved in Insulin receptor signaling in mouse 3T3-L1 adipocytes [41]. In this context, CAP functions as an adapter to recruit c-Cbl to the insulin receptor where it becomes activated by phosphorylation [45]. Furthermore, CAP was shown to bind to dynamin and to play a role in receptor mediated endocytosis in mammalian cells [46]. A different role was attributed to CAP in the formation of stress fibers and focal adhesions *in vitro* [47]. This was consistent with its interaction with Focal Adhesion Kinase, Vinculin and Paxillin [39, 43, 44, 47]. Vertebrate CAP is present in costameres and intercalated discs, both *in vitro* and in mouse muscle sections [48] and is prominently expressed in the murine and zebrafish heart ([47]; S. Abdelilah-Seyfried, University of Potsdam, personal communication). In *Drosophila*, CAP was first described for its interactions with proteins of the Wnt signaling pathway [49, 50]. More recently, Bharadwaj and coworkers [51] reported that CAP regulates two actin-rich structures: muscle attachment sites (MAS), which connect somatic muscles to the body walls, and scolopale cells required for mechanosensation. They also found, that the CAP-interacting protein Vinculin is required for CAP recruitment to MAS and that *vinculin* mutants partially phenocopy *CAP* mutations.

We demonstrate that CAP is required lifelong for the development and function of the *Drosophila* heart, in cooperation with the IAC. In migrating cardioblasts, *CAP* loss of function results in irregular cardioblast alignment and variation in cardioblast numbers, indicating a role in the regulation of the coherence of migrating cells. *CAP* genetically interacts with *scab* (encoding the αPS3-Integrin subunit), and *rhea* (encoding Talin), and *CAP* mutant hearts show a strongly reduced heart lumen due to defects in cell shape. In larval, pupal and adult hearts, CAP colocalizes with Integrin near Z-bands and at cell contact sites of contralateral cardiomyocytes. Adult *CAP*-mutant hearts exhibit local defects in cell contacts and myofibrillar organization which result in strongly reduced heart function.

## Materials and methods

### Fly stocks and genetics

The control strain used for all experiments was *w*^*1118*^. The fly stocks *scb*^*2*^, *rhea*^*1*^, *P{TRiP*.*HM05250}attP2* (*UAS-CAP RNAi*) and *Df(2R)BSC281* were obtained from the Bloomington stock center. The homozygous lethal *Df(2R)BSC281* (46D-47A) removes 3220 kbp DNA encoding *CAP* and 32 up- and downstream flanking protein coding genes. The FlyTrap GFP lines *P{PTT-GB}ZASP52*^*G00189*^ (*ZASP52-GFP*), *P{PTT-un1}sls*^*ZCL2144*^ (*Kettin-GFP*; [52, 53]) and *P{PTT-GA}CAP*^*CA06924*^ (*CAP-GFP*; [52, 54]) were provided by L. Cooley (Yale University, USA). The *handC3*.*1-GFP* line [55] was provided by A. Paululat (University Osnabrück, Germany). For tissue-specific RNAi knockdown, the driver lines *tinC+D-GAL4* and *twi*+*how*-*GAL4*, were generated by combining *tinCΔ4-GAL4* [56] with *tinD-GAL4* (=*TMEN6-GAL4 #52D1*; from J. Weiss, OHSU, Portland, USA; [57]) and *2xPE-twi-GAL4* [58] with *how*^*24B*^*-GAL4* [59], respectively. The *CAP*^*42b*^ and the *CAP*^*49e*^ mutant fly strains and *UAS-CAP-C* [51] were kindly provided by A. Kolodkin (Johns Hopkins University, Baltimore, USA). All experiments were carried out at 25°C except the RNAi knockdown experiments that were carried out at 29°C.

### Whole-mount *in situ* hybridizations

Whole-mount *in situ* hybridizations were performed as described by Lécuyer *et al*. [60]. Dig-labeled RNA sense and antisense probes were generated using the oligonucleotides CAP-fwd: ATGCCCAATAACCGCAATC and CAP-rev: TTTGGACTCGGTTCATTTTC. For probe detection Alkaline phophatase-coupled anti-Dig antibody (Roche Molecular Biochemicals) was used. Images were taken using Zeiss Axiophot microscope system with Nomarski optics.

### *Drosophila* CAP antiserum

To generate a Myc-His fusion protein, the DNA encoding the short CAP isoform CAP-PE/N with three SH3 domains (NP_001137639), was cloned into the pMH vector [61]. Purified Myc-His-CAP-PE/N was injected into rabbits for polyclonal antibody production (BioGenes GmbH, Berlin). The CAP antisera were affinity purified using the recombinant CAP-E/N protein.

### Immunoblotting

Protein extracts were isolated from 10 adult flies of the desired genotype. Shock frozen animals were homogenized in 50 μl 2× SDS sample buffer and boiled at 90°C for 10 min. Insoluble debris were removed by short centrifugation. For SDS-PAGE 10 μl of the resulting extracts per lane were used. The blot was blocked for 30 minutes with 10% dry milk powder (w/v) in phosphate buffered saline (PBS) containing 0.1% Triton X100 (PBT). Primary antibodies were diluted in 5% BSA in PBT and incubated overnight at 4°C. Antibodies used were rabbit anti-CAP (1:2.000, this study) and mouse anti-H2A (undiluted cell culture supernatant, monoclonal antibody Bx65; M. Frasch, H. Saumweber, unpublished). Alkaline phosphatase-coupled secondary antibodies (Dianova, Germany) were diluted 1:10.000 and phosphatase activity was visualized by colorimetric NBT/BCIP reaction.

### Immunohistochemistry and microscopy

Embryos collected from grape juice plates were dechorionated in 50% DanKlorix (Colgate-Palmolive), fixed for 30 minutes in a 1:1 (v/v) mixture of 4% formaldehyde in PBS and n-heptane and devitellinized by shaking in a 1:1 (v/v) mixture of methanol and n-heptane. Dorsal vessels from adult flies were dissected in 1xPBS and fixed for 1h with 3.7% formaldehyde in 1xPBS. Staining of fixed embryos was as described by Knirr *et al*. [62]. If necessary Tyramide Signal Amplification (TSA, as indicated below) was performed using biotinylated secondary antibodies in combination with the Vectastain ABC Kit (Vector Laboratories) and fluorescent Tyramide reagent (PerkinElmer Inc.). Preparation of adult heart still inside an open abdomen was as described by Vogler and Ocorr [63]. Fixation and staining of such hearts still in their abdomina was according to Molina and Cripps [22] with the following modifications: Fixation in 3.7% formaldehyde was for 30–60 min at 4°C, blocking was for 30 min in PBT containing 5% BSA. Following overnight reaction with primary antibodies at 4°C, samples were washed 3x 15 min in PBT and binding of secondary antibody was for 2 h at room temperature. F-actin was visualized by staining fixed tissue using TRITC-coupled Phalloidin (Sigma, 0.3 μg/ml) in PBS for 1h at room temperature. Following three 15 min washes in PBT abdominal preparations were mounted in 85% glycerol, 4% propylgallate. The following primary antibodies were used: monoclonal antibodies for βPS1-Integrin/CF.6G11 (1:10), Rhea/Talin E16B (1:20, TSA) and Slit/C555.6D (1:10) were obtained from DSHB-NIH, University of Iowa, anti-GFP 3E6 (1:1000) was from Invitrogen. In addition the following polyclonal antibodies were used: rabbit anti-CAP (1:1000, this study), guinea pig anti-Doc2+3 (1:300, TSA; [64]) and rabbit anti-Mef2 (1:750; kindly provided by H.T.Nguyen, University of Erlangen-Nürnberg). Secondary antibodies were obtained from Invitrogen and used in a dilution of 1:1000. Data were taken using a Deltavision deconvolution microscope system (Applied precision, USA) and processed using softworx and imageJ software. The significance of changes in the number of Mef2+ or Doc+ cardioblasts was evaluated with a 2-sided t-test with equal variance. Differences with p-scores<0.05 were considered as significant (p<0.05 (*); p<0.01 (**); p<0.001 (***); S1 File).

### Histological sections and electron microscopy

Late stage embryos of the desired genotype were prepared for TEM analysis essentially as described [65]. Following washing with 0.1 M cacodylate buffer, tissue was stained with 1% osmium tetroxide for 1 hour, dehydrated in a graded ethanol series and propylene oxide and embedded in Poly/BedR 812 (Polysciences, Inc., Eppelheim, Germany). Semi-thin cross sections of each 1 μm were made on a Leica Ultracut S ultramicrotome. Sections were stained with toluidine blue for 3 minutes at 70°C. Image acquisition was on a Zeiss Axiophot microscope system. Ultrathin sections were contrasted with uranyl acetate and lead citrate and examined with a FEI Morgagni electron microscope. Digital images were taken with a Morada CCD camera and the iTEM software (Olympus Soft Imaging Solutions GmbH, Münster, Germany).

### Live-imaging of larval heart beat

Live imaging was performed using *w*^*1118*^ control or *CAP*^*49e*^ mutant L3 larvae carrying the *handC3*.*1-GFP* reporter line [55]. Larvae were anesthetized for 3 minutes with Flynap^®^ (Carolina Biological Supply Company) according to manufacturer´s instructions and immobilized on double-faced scotch tape on microscopic slides. Following 10 minutes recovery, larvae were imaged immediately on a Deltavision microscope system. Data were recorded as single optical sections with 10 frames per sec (fps) and processed using softworx software (Applied precision, USA). Data were further processed as avi files using imageJ software. From stills of these files the minimal (DCN^min^) and maximal (DCN^max^) distance of CMC nuclei close to the inner valve cells were measured for each 10 cycles of heart beat per animal. Average and standard deviation was determined for the control (n = 3) and the *CAP*^49e^ mutant group of animals (n = 15). From these values percent of apparent fractional shortening (AFS) was determined according to: [(DCN^max^–DCN^min^)/DCN^max^] x 100%. Following data recording the larvae were carefully removed from the tape and checked for vitality. Data were graphically displayed and significance of the data was evaluated by 2-sided t-test for unequal variances (type 3). Differences with p-scores<0.05 were considered as significant (p<0.05 (*); p<0.01 (**); p<0.001 (***); S1 File).

### Dye angiography

For dye injections staged pharate adults (<90 h APF) were used. The preparation of flies, the injection process and the movie acquisition were described by Drechsler et al. [66]. Briefly, pharate adults were glued on a glass slide and the operculum was removed to allow recording of the dye accumulation. 1 μg/μl Uranin, a fluorescent tracer solution, was microinjected laterally into the abdomen of the animal. Dye accumulation in the head region was recorded for 180 sec by fluorescence stereomicroscopy with a digital camera at 10 frames/sec. Pixel intensities were measured within the region of interest (R.O.I.; see red outline in Fig 8b) in the fly head using ImageJ-Fiji software. The average from three consecutive frames (0.3 sec) was determined for each animal for a time interval of 180 sec. Background was removed from the data of each animal individually by subtracting the 0.3 sec interval value with the minimal value determined from all time points. The resulting values were graphically displayed for single animals and for the average of each genotype. The p-scores were determined by a two-sided t-test with unequal variance (type 3). Differences with p-scores<0.05 were considered as significant (p<0.05 (*); p<0.01 (**); p<0.001 (***); S1 File).

## Results

### *Drosophila* CAP is expressed in the dorsal vessel and is located at the periphery of cardioblasts

In a screen for new genes involved in *Drosophila* heart morphogenesis we found CAP to be expressed in the dorsal vessel (DV). Since CAP is conserved in evolution, and since the zebrafish and mouse CAP orthologues are highly expressed in the heart ([47]; S. Abdelilah-Seyfried, University Potsdam, unpublished), we decided to further explore the heart specific function of CAP in *Drosophila*.

**Fig 1:**
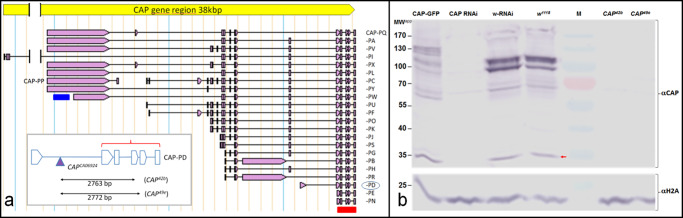
CAP gene structure and protein expression. (a) Exon-intron structure of the annotated CAP proteins [68]. Note that all but the CAP-PP coding transcript contain 5 common 3´ exons. The bars indicate the location of probes used for *in situ* hybridization (blue) and the protein isoform CAP-PE/N used for CAP antigen expression (red), respectively. **Inset:** indicates length and position of the deletions *CAP*^*42b*^ and *CAP*^*49e*^ (double arrows) and the insertion site of *CAP*^*CA06924*^ (used to generate the theses deletions) in relation to isoform CAP-PD (also encircled in the list of all annotated CAP proteins) according to [51]. The deletions remove two of the three SH3 domains (drawn as pentagons) common to all CAP isoforms except CAP-PP. The red bracket indicates the exon-intron structure of CAP PE/N isoform that was used for immunization. b) Western blot reacted with either rabbit anti-CAP (top) or anti-H2A (loading control below). The type of the adult fly extracts loaded is as indicated on top and specified in the text: CAP-GFP (lane 1); *w*^*1118*^; *UAS-CAP RNAi; da-GAL4* (lane 2); *w*^*1118*^; *UAS-white RNAi; da-GAL4* (lane 3); *w*^*1118*^ control strain (lane 4); protein molecular weight marker (lane 5); *CAP*^*42b*^; *hand-GFP* (lane 6), *CAP*^*49e*^; *hand-GFP* (lane 7). Red arrow points to the position of CAP isoform PN/E.

The *CAP* gene spans 38 kbp on chromosome 2R (46F9-47A1) encoding 23 alternate spliced transcripts that encode 20 unique polypeptides (Fig 1a; [67, 68]). To detect the CAP protein, we generated an antiserum against the short CAP isoform CAP-E/N, which consists of the three highly conserved SH3 domains (Fig 1a, Inset). According to the gene model this antiserum should detect all annotated CAP isoforms except CAP-PP (Fig 1a). On Western blots with cell extracts of adult flies (Fig 1b; for entire blot see S4 Fig) our CAP antiserum reacts with several (~17) polypeptides (Fig 1b, *w*^*1118*^ lane 4). This number corresponds to most of the 20 predicted CAP isoforms, as was reported previously for a similar CAP antiserum [51]. *CAP*^*42b*^ and *CAP*^*49e*^ mutant cell extracts show a weak nonspecific immunoreaction with CAP antiserum (Fig 1b, lanes 6 and 7). Extracts of animals with *da-GAL4*-induced *UAS-CAP RNAi* (see below) show a >90% reduction in signal intensity (Fig 1b, CAP RNAi lane 2 compare to. *w*^*1118*^, lane 4). Together, these results show that our antiserum is specific for CAP.

**Fig 2:**
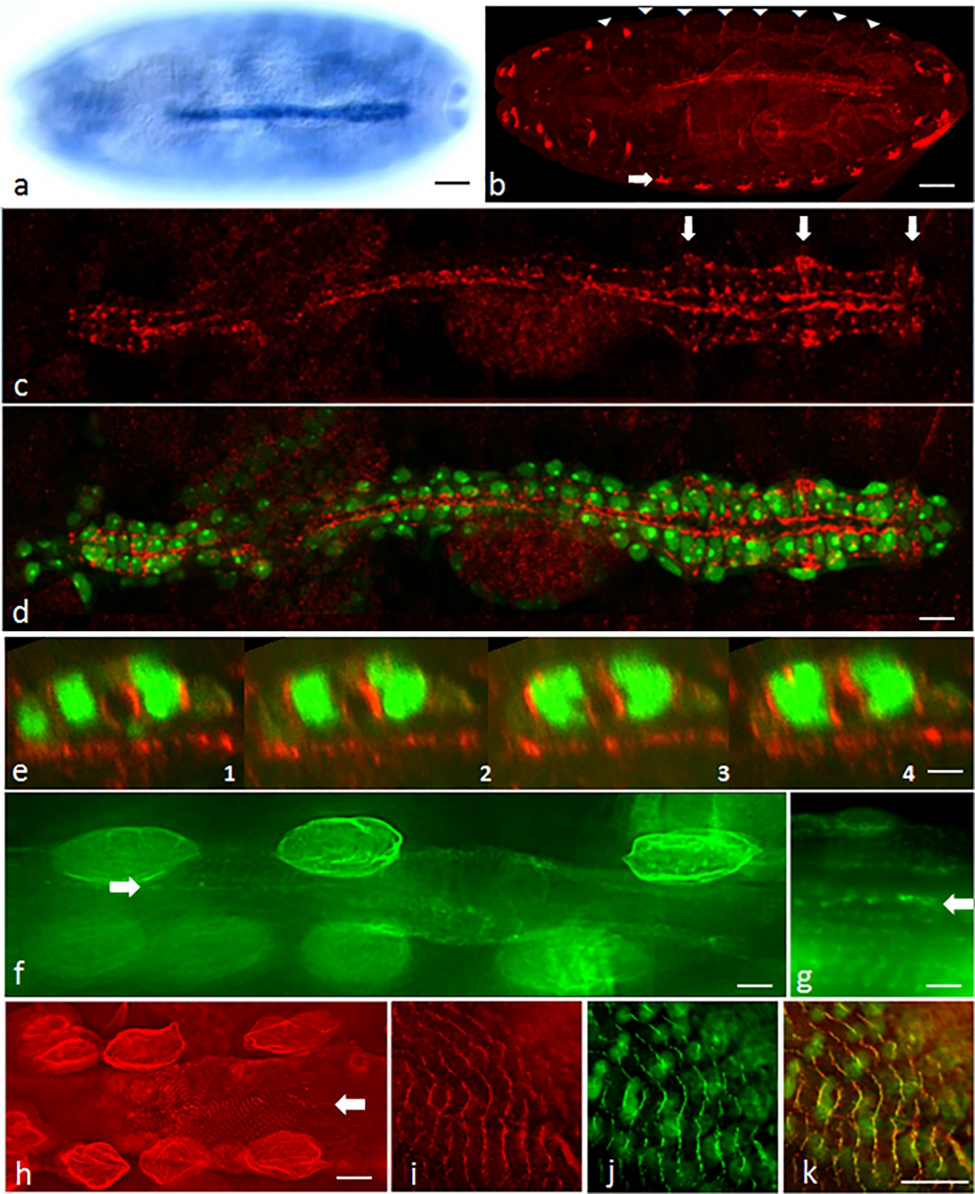
CAP is expressed in the *Drosophila* heart throughout development. (a) Dorsal view of a stage 16 embryo following *in situ* hybridization using the 5´ *CAP* probe shown in Fig 1a. (b) Optical section of a whole mount stage 16 embryo following anti-CAP staining; white arrowheads: muscle attachment sites; open arrow: lateral pentascolopidial chordotonal organs; bar in (a, b): 35 μm; (c-e) Dorsal vessel of a stage 16 embryo stained for expression of CAP (red); *hand*-GFP signal green; (c) Dorsal view projections of anti-CAP staining only; arrow: prominent CAP staining near the ostia; (d) Same as (c) with additional *hand*-GFP signal. CAP is present in CMCs, but absent from pericardial cells, labeled by *hand*-GFP only; bar: 10 μm. (e) Consecutive Z-sections of the dorsal vessel; longitudinal distance between two consecutive sections: 20 μm; bar: 5 μm. (f, g) Optical sections of L3 larval heart expressing CAP-GFP (green); Note the presence of CAP at circular myofibrils and contact sites of contralateral CMCs (open arrow); strong staining is also observed in larval pericardial cells; bar (f): 10 μm; (g): 5 μm. (h) Optical sections of adult heart; CAP antibody staining (red) of circular and longitudinal myofibrils and at contacts of contralateral myocardial cells (open arrow). Pericardial cells are prominently stained; bar: 15 μm. (i-k) anti-CAP staining together with Kettin-GFP fluorescence of adult heart at higher magnification. (i) CAP (red) and (j) Kettin (green) colocalize near Z-bands on circular myofibrils (k, merge); bars (i-k): 8 μm.

Next, we studied CAP expression in the *Drosophila* dorsal vessel (Fig 2, S1 Fig). By whole mount *in situ* hybridization using an N-terminal probe we find strong *CAP* expression in the DV of the *Drosophila* embryo (Fig 2a). The first expression of *CAP* is detected in embryos at stage 11 in the garland cell primordium (S1a Fig). Starting with stage 13/14 *CAP* expression is detected in the heart progenitors and the visceral mesoderm (S1b Fig). Thereafter *CAP* expression in the heart increases up to stage 16 (S1c–S1e Fig). Until stage 16/17 *CAP* expression in the heart is restricted to cardioblasts (CBs).

In embryos we find CAP protein in the scolopale cells, a component of the chordotonal organ (Fig 2b, open arrow), the muscle attachment sites (MAS; Fig 2b, arrowheads) and in the DV, in agreement with data published previously [51]. CAP immunostaining of CBs is initially weak but increases during heart development. In stage 16, CAP is present in all CBs of the DV (Fig 2c), but not in the pericardial cells (Fig 2d). CAP is distributed in a granular pattern at the CB periphery. It is enriched at the luminal and abluminal side but is also present in between ipsilateral cells. On the luminal side CAP forms a rather continuous layer compared to the granular staining on the abluminal side. This difference is more obvious in the heart region. Locally, we observe abluminal accumulations of granular CAP staining near the ostial cells (Fig 2c, open arrows). In Z-sections, we see the same principal features of luminal and abluminal staining. Of note, besides the luminal location, CAP staining often reaches into the dorsal contact zones of paired contralateral cells (Fig 2e). In the larval and adult heart CAP is localized at cell contacts of contralateral cardioblasts and in the circular myofibrils of cardiomyocytes (Fig 2f–2h; S2d, S2g and S2j Fig). In adult hearts, in addition, it is localized in ventral longitudinal myofibers (S2a and S2j Fig). In both types of myofibrils CAP staining localizes near Z-bands, as evident by Kettin and ZASP colocalization (Fig 2i–2k; S2a–S2l Fig). CAP most closely colocalizes with Integrin at contacts of contralateral cardiomyocytes (CMCs; S2m–S2o Fig). The strong CAP/Integrin colocalization along the circular myofibrils in these cells suggests that CAP is localized in costameres (S2m–S2o Fig; see Discussion). In contrast to their embryonic precursors, larval and adult pericardial cells show a strong ubiquitous CAP distribution (Fig 2f and 2h; S2j, S2l, S2p and S2r Fig). Altogether our analysis shows that CAP is continuously expressed in cardiac cells throughout *Drosophila* development.

### *CAP* mutants display abnormal cardioblast cell number and alignment

**Fig 3:**
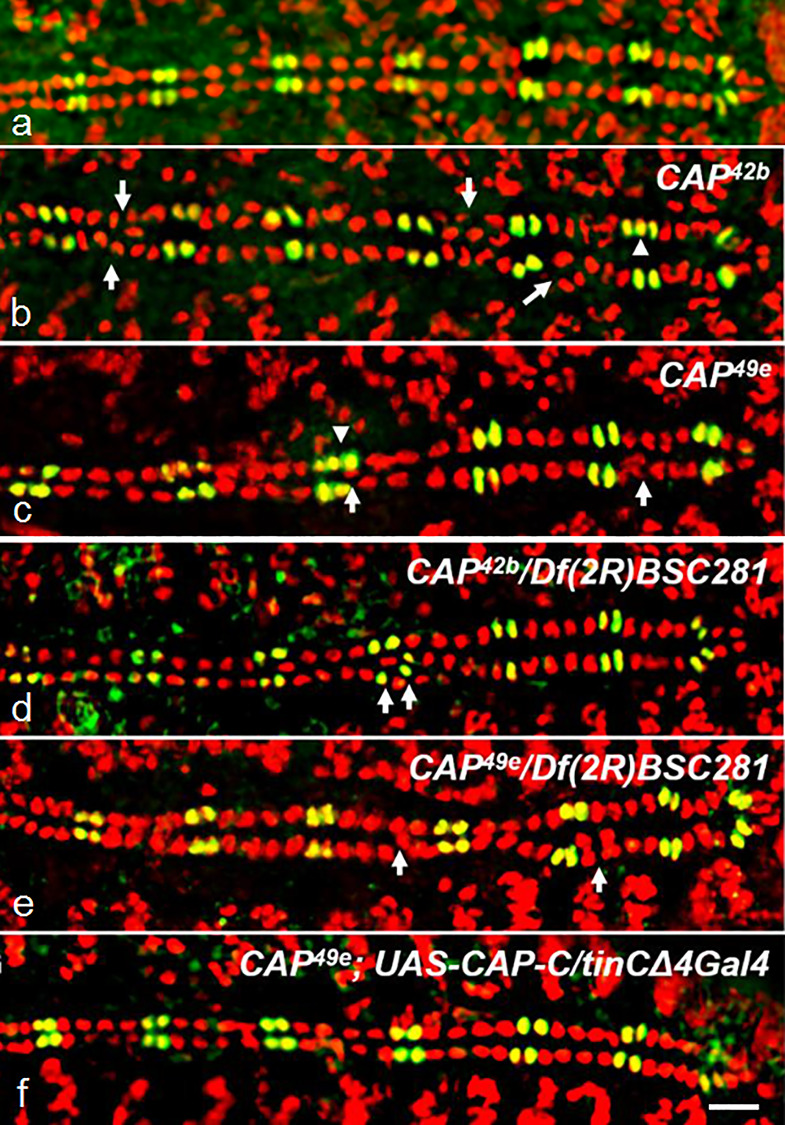
*CAP* mutants display abnormal cardioblast cell number and alignment. Optical sections of stage 16 whole mount embryos costained for Doc2+3 (green) and Mef2 (red); anterior left; arrows: amplification and/or internalization of CBs; arrowheads: amplification of Doc+Mef2 positive CBs. (a) *w*^*1118*^ *control* showing the typical pattern of 2 Mef2+Doc CBs (yellow) and 4 Mef2-only CBs (red) per hemisegment; (b) *CAP*^*42b*^, (c) *CAP*^*49e*^, (d) *CAP*^*42b*^*/Df(2R)BSC281*, (e) *CAP*^*49e*^*/Df(2R)BSC281*: amplification and internalization of Mef2 only and Mef2+Doc CBs is observed. (f) *CAP*^*42b*^;*UAS-CAP-C/tinCΔ4-GAL4* rescued embryo showing normal heart development (see Table 1); bar 10 μm.

To investigate the role of CAP during heart morphogenesis we used the molecularly characterized P-element excision mutants *CAP*^*49e*^ and *CAP*^*42b*^. Both mutants carry C-terminal deletions removing two of the three SH3-domain containing exons common to most annotated isoforms [51] except CAP-PP (see Fig 1a; inset). Western blot analysis and immunostaining indicates the absence of most CAP protein isoforms in these two alleles (Fig 1b). However, as mentioned, our antiserum is directed to the CAP C-terminus and therefore we cannot exclude the presence of isoform CAP-PP in these mutants (see Discussion). With this caveat in mind we studied the morphology of the DV of CAP mutants using anti-Mef2 antibodies, which stain all CB nuclei in combination with anti-Doc2+3 antibodies, which stain the ostial cell nuclei only (Fig 3).

In *w*^*1118*^ controls or heterozygous *CAP* mutants the CBs line up in two rows that pair exactly with their contralateral partners at the dorsal midline (Fig 3a). A segmental organization of the DV becomes apparent by molecular markers that is exactly matched between opposite CB rows. Wildtype type hemisegments contain two Doc/Svp-positive cells (stained for Mef2 and Doc) and four Tinman-positive cells (stained by anti-Mef2 only; Fig 3a). When homozygous, both *CAP* mutants show a DV phenotype characterized by irregularities in the alignment of CBs, blister formation, occlusion of CBs between the adjacent rows of cells and gain or loss of CBs. Instead of two contralateral CBs, often three cells are meeting at the dorsal midline, with one or more central CBs separating peripheral CBs (Fig 3b and 3c). In addition, *CAP* mutant DVs frequently contain additional CBs in several hemisegments and may have 6 to 8 Tinman-positive CBs per hemisegment (Fig 3b and 3c; Table 1). In a t-test of increased numbers of Mef2+ cells compared to *w*^*1118*^ the p-score for *CAP*^*42b*^ is p = 7.00E-09 (***) and for *CAP*^*49e*^ p = 2.98E-05 (***; S1 File). Occasionally we observed an increased number of Doc/Svp-positive CBs as well (Fig 3b and 3c). More rarely we also found hemisegments with less than 4 Tinman-positive CBs (arrows S3a Fig). The DV phenotype is already expressed during CB migration (S3a Fig).

**Table 1:** Quantification of the embryonic heart phenotype of *CAP* mutants, *UAS-CAP RNAi*, *CAP* mutant rescue and of *CAP* double heterozygous animals. Hearts are categorized as defective if displaced or misaligned Mef2-stained CB nuclei where observed.

Quantification of the CAP phenotype			
Genotype	Heart defects	Mef expressing cardioblasts	Doc expressing cardioblasts
*w*^*1118*^	0% (n = 52)	103.5 ± 1.2 (n = 22)	28,2 ± 0.7 (n = 22)
*CAP*^*42b*^	64% (n = 115)	107.5 ± 1.2 (n = 27)	28,4 ± 0.9 (n = 27)
*CAP*^*42b*^*/Df(2R)BSC281*	53% (n = 17)	106.3 ± 1.9 (n = 10)	28,4 ± 1.2 (n = 10)
*CAP*^*49e*^	63% (n = 75)	106.2 ± 2.6 (n = 29)	28,6 ± 0.9 (n = 29)
*CAP*^*49e*^*/Df(2R)BSC281*	55% (n = 20)	105.8 ± 2.9 (n = 8)	28,9 ± 1.5 (n = 8)
*Df(2R)BSC281*	72% (n = 18)	n.d.	n.d.
*UAS-CAP RNAi*	0% (n = 15)	104.0 ± 0 (n = 15)	28,2 ± 0.4 (n = 15)
*UAS-CAP RNAi; tinC+D-GAL4*	41% (n = 12)	104.0 ± 0 (n = 12)	28,0 ± 0.3 (n = 12)
*UAS-CAP RNAi; twi+how-GAL4*	64% (n = 14)	104.0 ± 1.0 (n = 14)	29,0 ± 1.0 (n = 14)
*CAP*^*49e*^;*UAS-CAP-C/tinCΔ4Gal4*	26% (n = 19)	104.3 ± 1.2 (n = 8)	28,3 ± 0.7 (n = 8)
*CAP*^*49e*^; *rhea1*	47% (n = 17)	105.7 ± 2.0 (n = 6)	28,2 ± 0.4 (n = 6)
*CAP*^*49e*^*/scb2*	48% (n = 29)	104.2 ± 1.1 (n = 11)	28,1 ± 0.3 (n = 11)

The DV phenotype was observed in 63% of *CAP*^*49e*^ and 64% of *CAP*^*42b*^ embryos (Table 1). However, it is variable in expression. In the *CAP* mutant embryos with recognizable aberrations, the phenotype ranges from embryos in which only one segment is affected to clearly abnormal hearts which feature more irregularities. We did not further subdivide recognizable phenotypes according to their strength. On the other end, some of the subtle defects may not have been recognized and thus classified as wildtype-like (see Discussion). Both *CAP* mutations heterozygous over *Df(2R)BSC281* show a similar DV phenotype (deviations in alignment and cell number) as the homozygous *CAP* mutants with a slightly reduced penetrance (Fig 3d and 3e; Table 1). However, in a t-test for the number of Mef positive CBs we see no significant difference between the *CAP*/*Df(2R)BSC281* heterozygotes and the corresponding homozygous *CAP* mutants (p-scores for *CAP*^*42b*^/*Df(2R)BSC281* are p = 0.1759 and for *CAP*^*49e*^/*Df(2R)BSC281* p = 0.6491; S1 File). Homozygous *Df(2R)BSC281* animals survive as stage 17 embryos and can unambiguously be identified by the absence of CAP or Nidogen (Ndg) staining (*Ndg*, is also removed by *Df(2R)BSC281*). 72% of *Df(2R)BSC281* homozygotes (n = 18) show a DV alignment phenotype (Table 1). Probably the reduced penetrance in this case is caused by maternal effects of heterozygous female parents, however, this was not tested. To further corroborate the *CAP* DV phenotype we depleted CAP by RNAi knockdown. The *UAS-CAP RNAi* construct induced by the ubiquitous driver *da-GAL4* strongly reduced CAP expression in adults (Fig 1b, lane 2) and induction of *UAS-CAP RNAi* generated a similar DV phenotype as described for *CAP* mutants. Following induction by *tinC+D-GAL4* 41% of the embryos show this phenotype. Induction by *twi+how-Gal-4* produces a DV phenotype in 64% of randomly tested embryos. However, the number of Mef2+ CBs is closer to *w*^*1118*^ (S3 Fig; Table 1). The phenotypic effects of *CAP*^*49e*^ and *CAP*^*42b*^ are partially (~50%) suppressed by overexpressing the CAP-PC isoform via the CB -specific driver *tinCΔ4-Gal-4* (Fig 3f, Table 1). Under rescue conditions (*CAP*^*49e*^;*UAS-CAP-C/tinCΔ4Gal4*) the number of Mef2+ CBs is closer to the *w*^*1118*^ than with *CAP*^*49e*^ (p-score *CAP*^*49e*^;*UAS-CAP-C/tinCΔ4Gal4* p = 0.0956 and *CAP*^*49e*^ p = 2.9807E-05; S1 File).

### CAP is required for heart lumen formation

**Fig 4:**
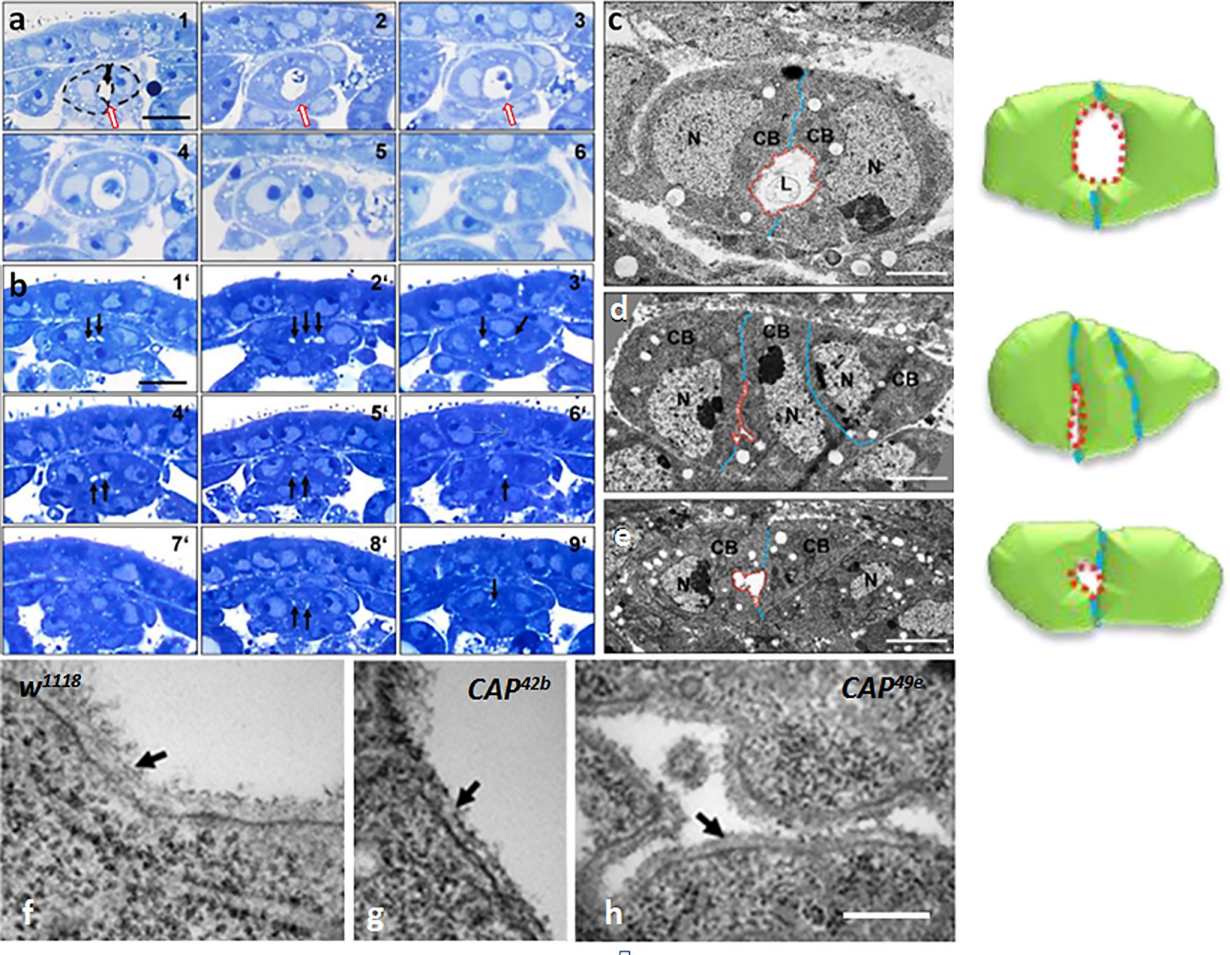
Heart lumen formation is disrupted in *CAP* mutants but basal membranes still form. (a, b) Light microscopy of serial semi-thin sections through the dorsal vessel of stage 16 embryos stained by toluidine blue. (a) *w*^*1118*^ embryo (six different cut positions, a1-6). Two crescent shaped contralateral cardioblasts (dashed lines in A1) with light blue nuclei and darker blue cytoplasm encircle the white lumen. (b) In a *CAP*^*49e*^ embryo (b1´-9´) the lumen (labeled by black arrows) is much smaller, often split in two or three minilumina or absent altogether (b7´); sometimes three CBs are involved; bar 10 μm. (c-e) Transmission EM of ultrathin sections of the heart region. (c) *w*^*1118*^ embryos; the luminal boundary between the two crescent shaped contralateral CBs is labeled red, their contact sites are labeled blue. (d) *CAP*^*49e*^; in the example shown three non-crescent formed CBs come together resulting in an extremely small lumen. (e) *CAP*^*42b*^; in the example shown only the left CB shows crescent formation. The right one does not, resulting in a significant reduction of the luminal space; bar: 2 μm. The cartoons to the right of c-e schematically illustrate the principal features shown of the TEM images. (f-h) Transmission EM of ultrathin sections of an embryo stage 16/17 showing CB luminal regions in controls and *CAP* mutants. (f) In the *w*^*1118*^ control the basal membrane at the luminal domain is marked (black arrow). An apparently normal basal membrane (arrows) is formed in (g) *CAP*^*42b*^ and (h) *CAP*^*49e*^; bar in (h): 200 nm.

Next, we asked whether a normal heart lumen is formed in *CAP* mutants (Fig 4). To compare the ultrastructure of the CBs in normal and mutant DVs in terms of size, shape and lumen formation, we analyzed wild type and *CAP* mutant embryos on serial semi-thin cross sections by light microscopy (Fig 4a) and ultrathin sections by TEM microscopy (Fig 4b). In *w*^*1118*^ control animals the paired contralateral CBs are sealed at the dorsal and ventral junctional region and the intercellular space remaining between these connections establishes a DV lumen (Fig 4a, arrow). In contrast, about 60% of *CAP* mutant embryos reveal a defect in lumen formation. In both *CAP*^*49e*^ and *CAP*^*42b*^ mutants, the CBs meet each other normally at the dorsal midline, but form a slit-like lumen or several split lumina, both much smaller than in the control (Fig 4b; arrows). Serial semi-thin cross sections show that the lumen phenotype is consistent along the length of the aorta and the heart proper region (Fig 4a and 4b). The lumen defects are not exclusively connected to misplaced CBs since they also occur in areas with just two contralateral CBs (see Fig 4e). As evident in high resolution TEM, the typical crescent like shape of the CBs is distorted in *CAP* mutant animals. Especially the curved luminal domain seen in the controls seems to be disturbed. The cells have a more half moon-like appearance and enclose a slit-like lumen, as is emphasized in the corresponding cartoons (compare Fig 4c to 4d and 4e). Among the factors that affect CB shape and lumen formation are components of the basal membrane as well as molecules that guide CB polarization or organize membrane domains and their attachment to the cytoskeleton. According to our TEM data, CAP appears to be largely dispensable for basal membrane formation since an apparently normal basal membrane ensheaths the lumen of the DV in *CAP* mutants (Fig 4f–4h).

### CAP is required for fine tuning the localization of Slit and Integrin at the luminal domain

**Fig 5:**
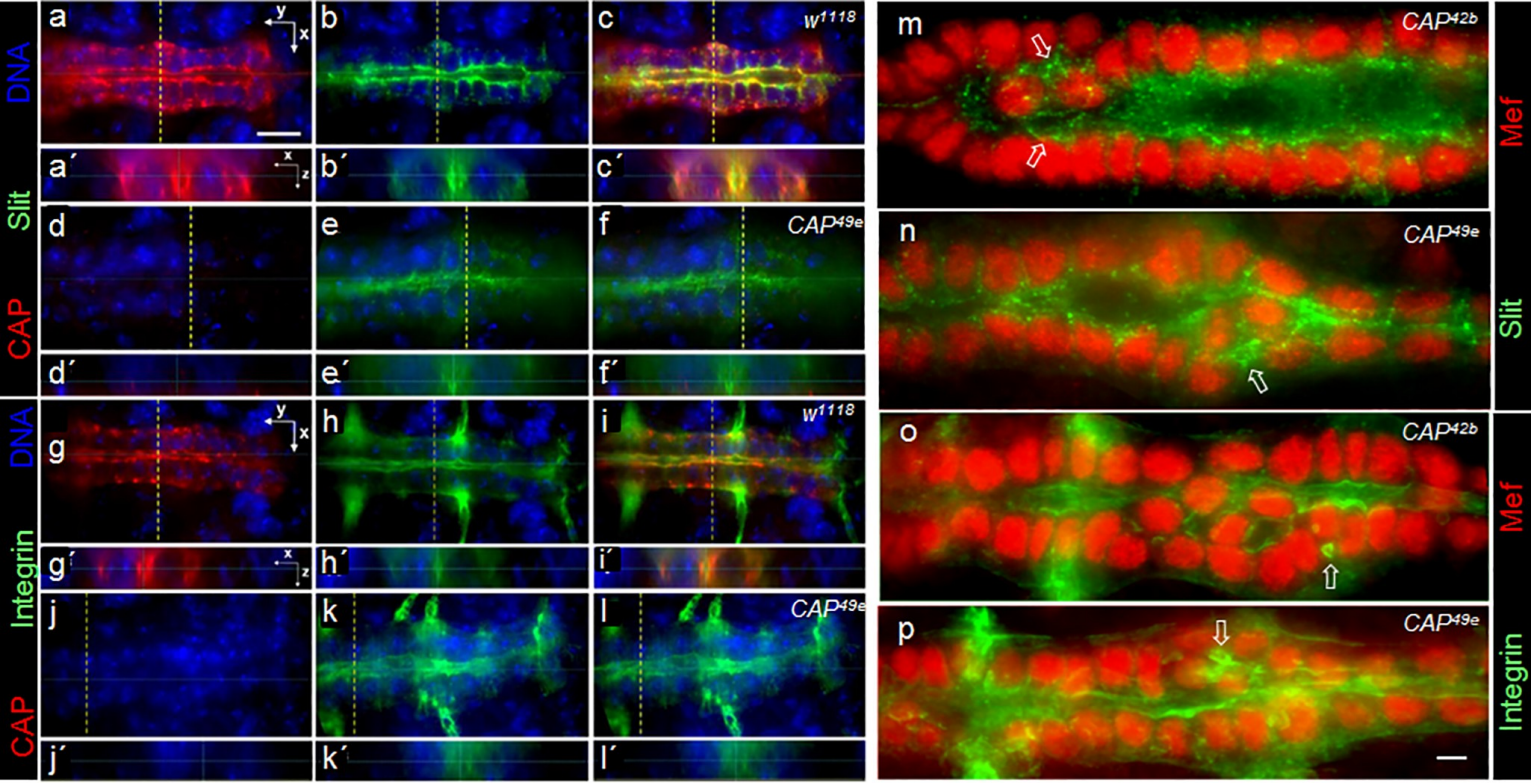
CAP is required for establishment of a distinct luminal boundary. Optical sections of the heart region of stage 16 whole mount embryos stained for CAP (red) and Slit (green) in (a-f) or CAP (red) and ßPS1-Integrin (green) in (g-l); DNA in blue. (a-c) *w*^*1118*^ control: CAP colocalizes with Slit at a clearly defined luminal boundary. (a´-c´) shows the heart lumen in Z-sections taken at the dashed lines in corresponding XY images. (d-f) *CAP*^*49e*^: The lumen is much smaller, with an ill-defined Slit stained boundary. (e´, f´) In Z-sections a lumen is hardly detectable. (g-i) *w*^*1118*^: CAP colocalizes with ßPS1-Integrin at the abluminal side and at a clearly defined luminal boundary. (j-l) *CAP*^*49e*^: The ßPS1-Integrin stained boundary towards the lumen looks ill defined. (k´, l´) A lumen is hardly detectable in Z-sections. anterior left; bar in (a): 10 μm. (m-p) Optical sections of the heart region stained by (m, n) Mef2 (red) and Slit antibodies (green) or (o, p) Mef2 (red) and ßPS1-Integrin antibodies (green). (m) *CAP*^*42b*^; note the ill-defined, granular Slit staining at the luminal cell boundary. On both sides of the central CBs minilumina are formed (arrows). (n) *CAP*^*49e*^: in the region of central CBs, Slit extensively spreads and encircles minilumina (arrow). (o) *CAP*^*42b*^: in the region of central CBs, the formation of a continuous lumen is obscured and ßPS1-Integrin is detected in extra luminal and miniluminal boundaries (arrow). (p) *CAP*^*49e*^: ill-defined ßPS1-Integrin distribution similar to (o); extra lumina form between peripheral and central CBs (arrow); bar in (p): 5 μm.

To find out whether CB polarization or the distribution of typical luminal marker proteins depends on CAP, we first analyzed the distribution of Slit and βPS1-Integrin in *w*^*1118*^ controls and *CAP*^*49e*^ mutants (Fig 5). In controls the CAP protein is localized to the luminal, abluminal and lateral membranes (Fig 5a and 5g) and the luminal marker Slit is localized predominantly at the luminal domain of the CBs (Fig 5b). Luminal Slit is co-localized with CAP protein (Fig 5c). In *CAP*^*49e*^ mutants no CAP protein can be detected (Fig 5d). Although Slit is still polarized and detected at the luminal domain of *CAP*^*49e*^ CBs, its distribution is less distinct than in the control. As a consequence the boundaries towards the lumen become ill defined (Fig 5e and 5f). In Z-sections, the lumen, if visible at all, appears to be much smaller than in the controls (Fig 5e´ and 5f´). βPS1-Integrin in the control localizes to the luminal and abluminal membrane domains of CBs and is co-localized with CAP at these sites (Fig 5h and 5i). *CAP*^*49e*^ mutants still display a polarized βPS1-Integrin localization, but similar to Slit, its distribution looks ill defined and the boundaries of the lumen are less well defined in comparison to the controls (Fig 5k and 5l). Co-staining of Slit and anti-βPS1-Integrin in the two *CAP* mutants with anti-Mef2 shows that the disruption of Slit and Integrin localization is stronger when associated with misaligned CBs. In such regions, Slit spreads into the basolateral space between peripheral CBs and around the central CBs as granular material that occasionally forms extra minilumina (Fig 5m and 5n). Similar spreading and minilumen formation associated with central CBs is also observed for ßPS1-Integrin (Fig 5o and 5p). This irregular organization often spreads along the heart tube from the misaligned CBs into immediate adjacent regions (Fig 5n and 5p).

### *CAP* genetically interacts with the integrin signaling components *scab* and *rhea* during heart development

**Fig 6:**
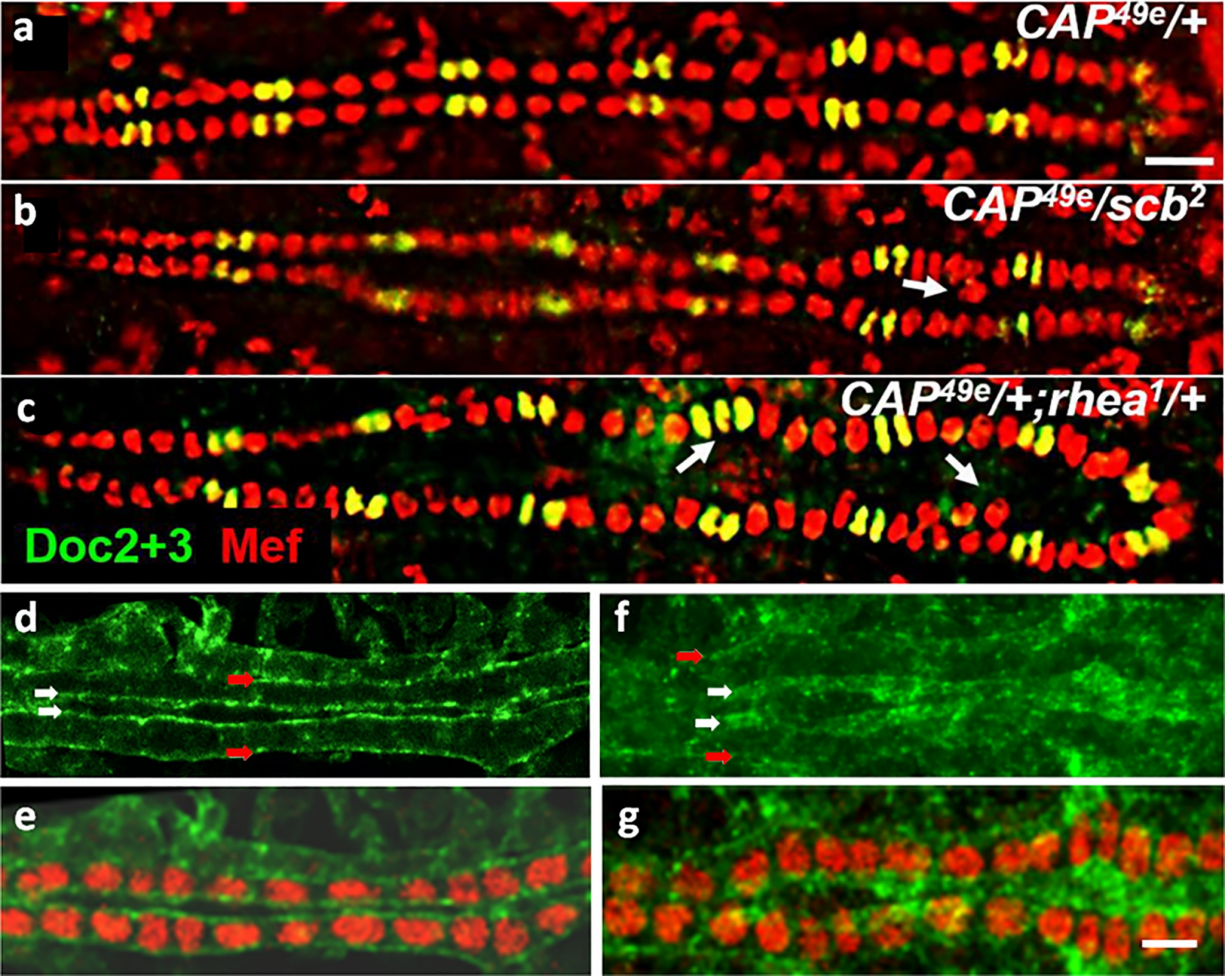
*CAP* genetically interacts with *scab* and *rhea*. (a-c) *CAP*^*49e*^ double heterozygote animals show the *CAP* phenotype. Optical sections of the heart region of stage 16 whole mount embryos costained with Mef2 (red) and Doc2+3 antisera (green). (a) *CAP*^*49e*^*/+* control; (b) *CAP*^*49e*^*/scab*^*2*^ with an additional central cardioblast in the heart region (arrow); (c) *CAP*^*49e*^*/+; rhea*^*1*^*/+* with 5 peripheral and 3 additional central cardioblasts and three ostial cells in the indicated heart regions. Anterior left; bar: 10 μm. (d-g) Optical sections of the dorsal vessel of stage 16 whole mount embryos costained with Mef2 (red) and Rhea/Talin (green) antibodies. (d) The *w*^*1118*^ control shows a distinct localization of Talin in the luminal and abluminal (open arrow) region with a clearly defined lumen (filled arrow). (e) same, costained with anti-Mef2. (f) In *CAP*^*49e*^ the Talin distribution at the luminal boundary is less well defined than in the control (filled arrow) and in the abluminal domain of the dorsal vessel Talin staining is also less distinct (open arrows). (g) Same costained with anti-Mef2. There are no internal extra cardioblasts in this section. Anterior left; bar 10 μm.

A recent study on CAP function implicates that CAP is acting downstream of Integrin signaling at muscle attachment sites and interacts with Vinculin [51]. Due to the co-localization of CAP and βPS1-Integrin in the *Drosophila* DV we asked whether CAP genetically interacts with Integrin signaling components during heart development (Fig 6). Previous studies reported that heterozygous *scab* and *rhea* mutants show normal DV development [18]. Heterozygous *CAP*^*49e*^ mutants also have a normal DV morphology (Fig 6a). Interestingly, double heterozygous animals, with *CAP*^*49e*^ either combined with *scab*^*2*^ or *rhea*^*1*^ show defects similar to those seen in *CAP* homozygous mutants (Fig 6). 48% of randomly selected *scab*^*2*^*/CAP*^*49e*^ double heterozygous embryos show a DV phenotype (Fig 6b; Table 1). In the case of *rhea*^*1*^*/CAP*^*49e*^ embryos 47% show a DV phenotype (Fig 6c; Table 1). This demonstrates that *CAP* genetically interacts with *scab* and *rhea* during heart development. As for Integrins, this observation is corroborated by our finding that Rhea/Talin localization is disrupted in a homozygous *CAP* mutant background. In control animals, Talin is localized at the luminal and abluminal periphery of CBs, demarcating a clearly defined cytoplasmic space (Fig 6d and 6e). In *CAP*^*49e*^ mutant animals the luminal Talin staining is similarly ill defined as was observed for Slit and ßPS1-Integrin in *CAP* mutants (Fig 6f and 6g). On the abluminal domain Talin distribution is also altered and less distinct.

### *CAP* mutant larvae and adults show an impaired heart function

**Fig 7:**
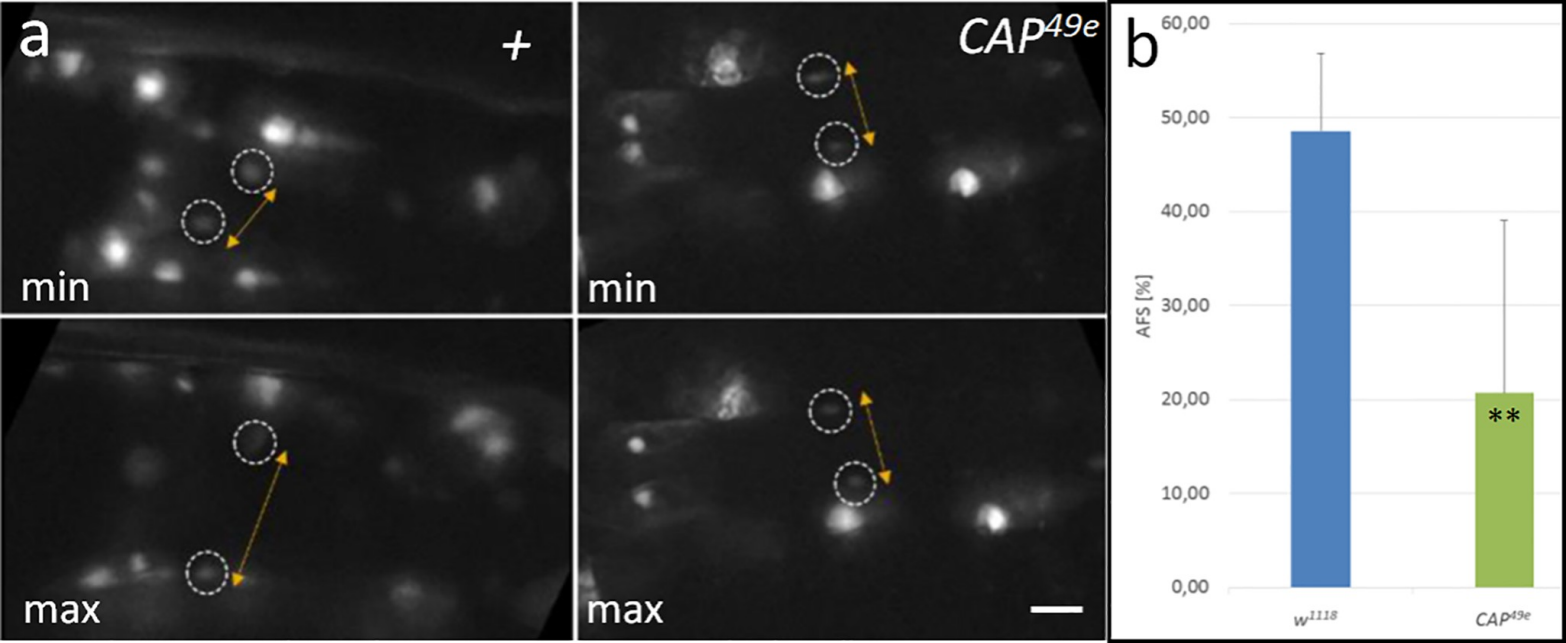
CAP is required for function of larval hearts. (a, b) Test for changes of apparent fractional shortening in L3 larvae. (a) *hand-GFP* controls (left panels, labeled +): stills of live imaging movies (see S1 Movie) are displayed and the distance of contralateral inner valve cell nuclei (encircled) at maximal extension (max) and minimal extension (min) is indicated by double arrows. (b) *CAP*^*49e*^ (right panels): stills of live imaging movies (see S2 Movie). Due to reduced valve opening, phenotypically mutant *CAP*^*49e*^;* hand-GFP* larvae show only a small difference in nuclear distance between the two states; bar: 10 μm (c) The average and standard deviation of the apparent fractional shortening (AFS; see Materials and methods) for the *hand-GFP* control (n = 3) and *hand-GFP; CAP*^*49e*^ mutants (n = 15) are displayed in the graph. The difference is significant (p = 0.0044¸**).

We argued, that the defects we have seen in *CAP* mutant embryos would affect the heart function later on. Therefore, we assayed the heart function in two different ways. First we checked for the heart contractility by performing live imaging of L3 larvae carrying the cardiac *handC3*.*1-GFP* reporter. Using this reporter, cycles of heart contraction and expansion can be observed and recorded in living larvae by the movement of CMC nuclei. We used the region immediately posterior to the internal valve to record the contraction cycles of the posterior heart as shown for the *w*^*1118*^ control (Fig 7a, S1 Movie). By measuring the distance of nuclei of contralateral CMCs from stills of the movies, the heart diameter at maximal extension, DCN^max^ (Fig 7a, top) and minimal extension DCN^min^ (Fig 7a, bottom) was determined and used to calculate the percentage of apparent fractional shortening (AFS% = [(DCN^max^–DCN^min^)/DCN^max^] x 100%; for details see Materials and methods). In control animals (n = 3) we observe an average fractional shortening of 48.6 ± 8% (AFS ranging from 39.2 to 54.6%). By contrast, in *CAP*^*49e*^ mutant larvae (n = 15) the CMC extension is significantly reduced (Fig 7b; S2 Movie), resulting in an AFS of 20.7 ± 18.3% for these animals (Fig 7c; ranging from 0.3 to 54.3%). Data analysis by t-test demonstrates that the difference between the controls and *CAP* mutants is statistically significant (p = 0.0044 (**); S1 File). Most of the mutant larvae display a clear reduction of the AFS with five out of 15 *CAP*^*49e*^ larvae featuring less than 5% of the average of the controls and eight larvae feature intermediate AFS phenotypes (see S1 File for details). Three out of 15 *CAP*^*49e*^ larvae (20%) exhibit an AFS in the range of the *w*^*1118*^ controls consistent with the incomplete penetrance of the phenotype in the DV of embryos.

**Fig 8:**
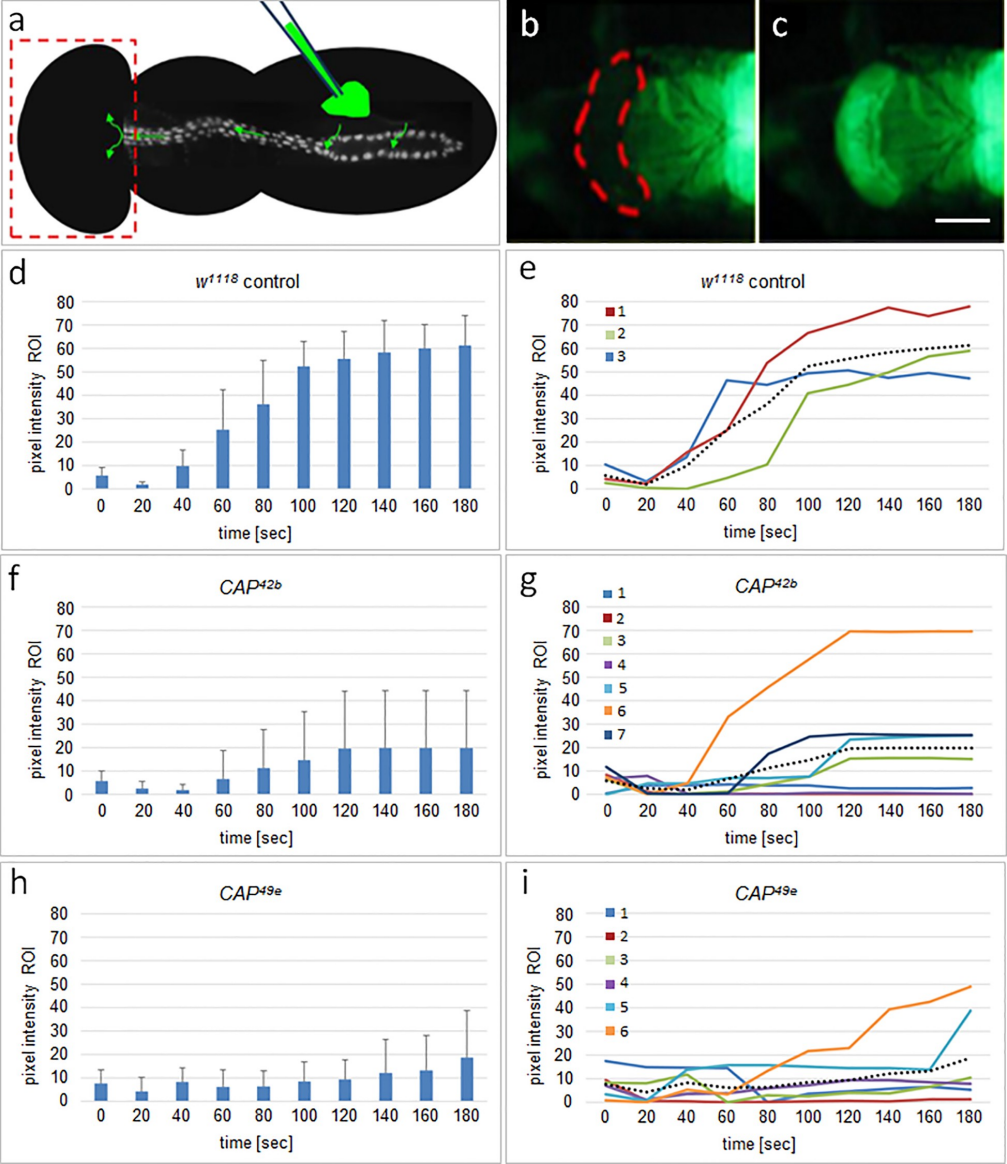
CAP is required for proper adult heart function. (a) Illustration of the dye angiography assay to analyze adult heart function: Fluorescent dye is injected into the abdominal region of pharate adults as indicated, taken up by the heart, pumped anteriorly and delivered into the head region (boxed). Transport efficiency was quantified by integration of fluorescence intensity in the ROI of the head region (outlined in b) at different time points. (b) Snapshot of the head region of a *w*^*1118*^ control at time of injection. ROI is indicated by dashed red line. (c) Same region 180 seconds after injection. (d, f, h) Graphs showing the average pixel intensity in the R.O.I. with corresponding standard deviation at different time-points following injection for (d) *w*^*1118*^ controls (n = 3), and with significantly lower values for (f) homozygote *CAP*^*42b*^ animals (n = 7, p = 0.0072; **) and (h) homozygote *CAP*^*49e*^ animals (n = 6, p = 0.0039; **). The corresponding graphs in (e, g, i) show the time course of transport of single animals as differently color coded solid lines for *w*^*1118*^ (1–3), *CAP*^*42b*^ (1–7) and *CAP*^*49e*^ (1–6); dashed lines in these graphs represent the average for each genotype. See text and S1 File for details.

Our second assay examined the adult heart function after the heart remodeling at metamorphosis. For this assay we injected a fluorescent dye as a tracer into the abdomen of late pharate adults (Fig 8a; [66]). Due to active pumping by the heart, the dye accumulates with time in the head region of the flies, which can be observed as an increase of the fluorescence emission in a defined region of interest (ROI), as seen for a *w*^*1118*^ control in Fig 8b and 8c. By integration at defined time intervals and calculating the average pixel intensity, the accumulation of the tracer in the ROI is quantitated and displayed graphically for single flies as seen for three control flies (Fig 8e). The average and standard deviation for the group are plotted separately in Fig 8d. The same data is displayed for *CAP*^*42b*^ and *CAP*^*49e*^ mutants (Fig 8f–8i). Compared to the control at 180 sec, with an average pixel intensity of 61.3 (n = 3), the transport efficiency of *CAP*^*42b*^ (average pixel intensity 19.8; n = 7) and *CAP*^*49e*^ (average pixel intensity 18.8; n = 6) is strongly reduced. The differences between the *w*^*1118*^ control and the *CAP* mutants are statistically significant (p = 0.0072 for *CAP*^*42b*^ (**) and p = 0.0039 (**) for *CAP*^*49e*^; see S1 File). However, considering single animals we observe differences in transport capability between tested animals. For instance, animals *CAP*^*42b*^ #2 and *#*4 had almost lost activity, whereas *CAP*^*42b*^ #3, #5, and #7 showed an intermediate phenotype, and *CAP*^*42b*^ #6 behaved like wild type (Fig 8g). A similar variation in phenotypic strength is also seen with *CAP*^*49e*^ (Fig 1i). This observation is in agreement with the variability of phenotypes observed in embryonic and larval *CAP* mutant hearts.

### CAP mutants show local aberrations in the morphology of the adult heart tube

**Fig 9:**
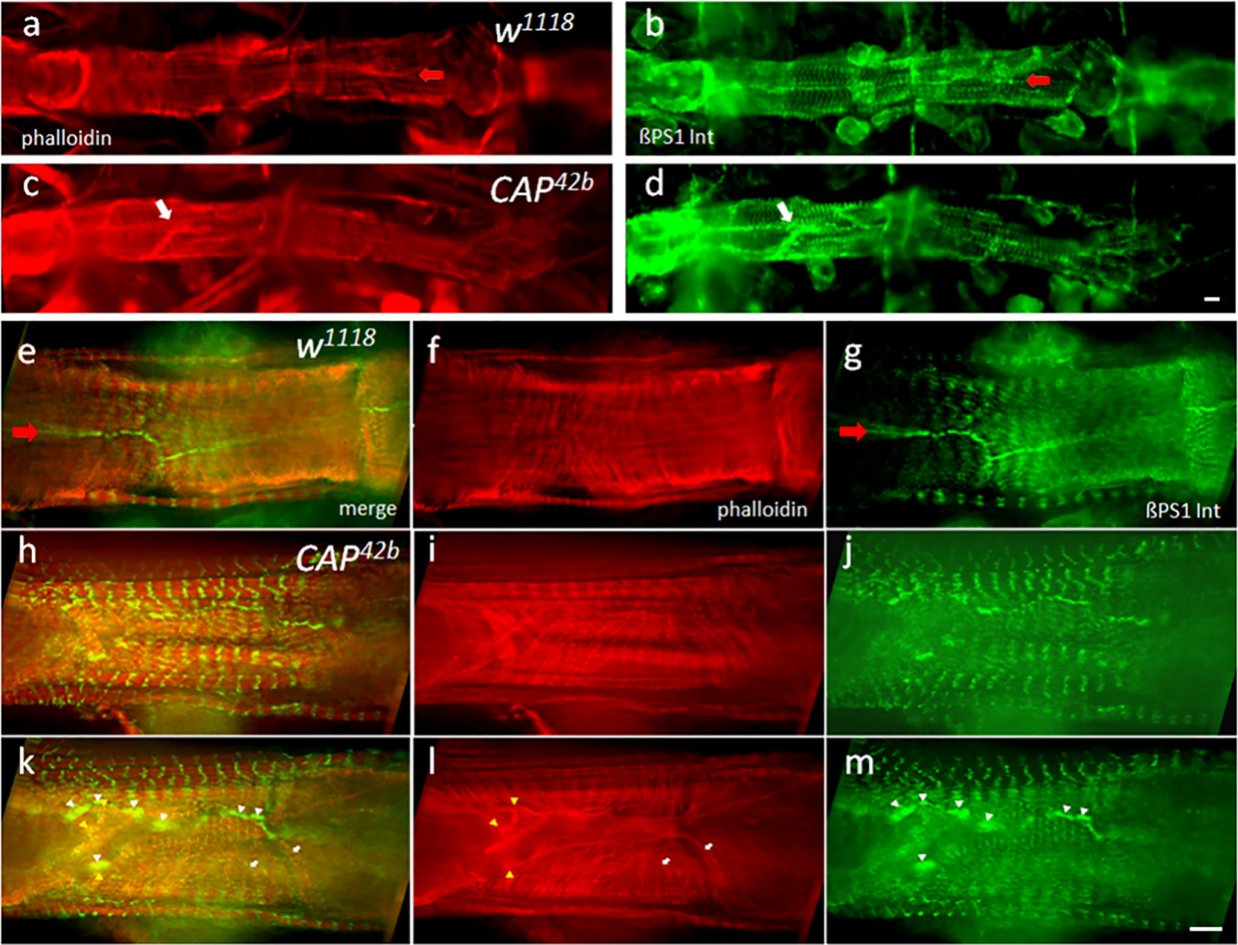
*CAP* mutants show local disruptions of adult heart morphology. Stacks of optical sections of adult hearts costained by F-actin/phalloidin and ßPS1-Integrin antibodies. (a, b) low magnification view of a *w*^*1118*^ control heart stained for (a) F-actin by phalloidin (red) and (b) ßPS1-Integrin (green). (c, d) low magnification view of a CAP^42b^ heart stained for (c) F-actin and (d) ßPS1-Integrin; note disruptions indicated by local accumulation of actin fibers and clusters of Integrin (white arrows in c and d, respectively); bar 8 μm. (e-m) higher magnification view of heart regions stained for F-actin (f, i, l) and anti-ßPS1-Integrin (g, j, m); (e, h, k) merge. (e-g) shows a projection of an image stack for the *w*^*1118*^ control with regularly arranged circular myofibrils (f; F-actin) that appear to smoothly continue across the line of contact sites of contralateral cells (open arrow in e and g; ßPS1-Integrin). (h-m) shows two consecutive projections of image stacks of a *CAP*^*42b*^ heart with cytological defects: (i, l) Disorganized cell contacts indicated by unorganized local masses and clustering of actin fibers (yellow arrowheads in l). Myofibrils in this region often do not contact the cell contact site and/or fold back into the cell interior (white arrows in l); (j, m) discontinuous, patchy organization of ßPS1-Integrin at cell contact sites (white arrowheads in m). (k) Unorganized actin masses and Integrin patches often colocalize (white and yellow arrowheads). bar: 8 μm.

Normally CAP is colocalized with ßPS1-Integrin near Z-bands/costameres and cell contacts between contralateral cardiomyocytes (S2m–S2o Fig). Therefore we wondered whether the observed impaired heart function in *CAP* mutants results from changes in the adult heart structure, as a consequence of the disruption of Integrin adhesion complex (IAC; Fig 9). In contrast to the *w*^*1118*^ control, where the contralateral cardiomyocytes (CMCs) form a continuous spiral pattern of myofibrils stained for F-actin by phalloidin (Fig 9a) and a smooth line of cell attachment sites stained by Integrin (Fig 9b, open arrow), *CAP* mutant hearts often exhibit local structural aberrations with clusters of actin and Integrin elements (Fig 9c and 9d, white arrows). Optical sections of the *w*^*1118*^ control at higher magnification (Fig 9e–9g) reveal the regular arrangement CMCs with spiral myofibrils (Fig 9e and 9f) that line up at cell contact sites (Fig 9e and 9g) and are continued by matching myofibrils in the contralateral cell. A smoothly stained line of ßPS1-Integrin demarcates these cell contact sites between contralateral CMCs. Integrin is also found at the costameres of circular and longitudinal myofibrils (Fig 9g). In the *CAP*^*42b*^ mutant (Fig 9h–9m) we do not observe obvious general defects in the Z-band architecture and costamere formation of myofibrils of CMCs. However, locally we detect aberrant, misaligned CMCs with a disrupted organization of βPS1-Integrin at cell contact sites (Fig 9j and 9m). In contrast to the controls, ßPS1-Integrin is found in such cells in clusters of increased Integrin accumulation (white arrowheads in Fig 9m), interrupted by sections that appear devoid of the protein. Interestingly, amorphous actin accumulations (Fig 9i and 9l) often form near such integrin clusters (yellow arrowheads in Fig 9l). Furthermore, *CAP*^*42b*^ mutants feature aberrant CMCs in which the myofibrils fail to align in register and often do not attach at cell contact sites (Fig 9i and 9l). Instead they bend back into the cytoplasm or run for a while parallel to the long axis of the heart (white arrows in Fig 9l), or multiple myofibrils or amorphous actin fibers initiate from ßPS1-Integrin clusters in the mutant (yellow arrowheads in 9l). Together, the Integrin mislocalization correlates with a disturbed architecture of the circular myofibrils and a discontinuity in their arrangement across contralateral CMCs (Fig 9k). These local defects at cell contact sites could break the structural continuity between contralateral CMCs that is important to transmit coordinated contraction.

## Discussion

As mentioned in the introduction, CAP has been reported to function in a variety of cell types. The data presented in our paper provide evidence that CAP plays a role in the robustness of *Drosophila* heart development and function. *CAP* mutant embryos show a DV phenotype characterized by local abnormalities in the alignment and numbers of CBs (often manifesting in luminal location of one or several CBs following closure of the dorsal vessel) as well as defects in lumen formation. Taken together with the protein localization data, this implies that CAP functions in processes near or within the membrane of cardiac cells. We suggest that the most important part of cardiac CAP function is related to the Integrin Adhesion Complex (IAC) for the following arguments. Apart from our observation that *CAP* mutants do not display breaks in the continuity of adjacent CBs, the DV phenotype is similar to that of homozygous *slit*, *integrin*, and *talin* mutants, double heterozygous mutant combinations among themselves or combinations with mutations in other Integrin-signaling components ([18, 27], and this work). This includes “clumps” [27] which we interpret as sites of misaligned CBs often forced into the DV lumen (see Fig 1H in [18]). In stage 15–16 embryos CAP colocalizes with ßPS1-Integrin (this work, Fig 5g–5i) and overlaps with Talin at the CB periphery [38]. Furthermore, we observed aberrant DV phenotypes, and hence a genetic interaction, in embryos double heterozygous for either *CAP*^*49e*^ and the αPS3-Integrin-encoding *scb*^*2*^ or *CAP*^*49e*^ and the Talin allele *rhea*^*1*^. The interaction with Scab may be cell non-autonomous. CAP/Vinculin interactions were documented *in vitro* [44] and more recently genetically in *Drosophila* muscle attachment sites *in vivo* [51]. We propose that CAP contributes to Integrin-actin cytoskeletal interaction to stably link the chain of CBs by cell adhesions and by adhesion to a common extracellular matrix (ECM). Local weakening or improper regulation of adhesiveness, possibly through a failure to transmit adhesion related forces or to coordinate organization of membrane microdomains, could result in mispositioning of migrating CBs relative to their neighbors.

The effect of the two *CAP* alleles used is not fully penetrant and is expressed as a series of intermediate states that differ in strength between individuals. Both alleles are small deletions removing two of the three SH3 domains common to all CAP isoforms except CAP-PP (Fig 1a inset; [51]). Bharadwaj and colleagues [51] suggest that both alleles likely result in a complete loss of CAP protein (amorphic type). We favor the idea that *CAP*^*42b*^ and *CAP*^*49e*^ are of non-amorphic type, consistent with the phenotypic effect observed. Although the deletions in both alleles affect the C-terminus of most isoforms, except CAP-PP, they leave their N-terminal portions intact, which, like CAP-PP, might have residual activity. In addition to CAP-PP, C-terminally truncated isoforms could be variably expressed in the *CAP42b* and *CAP49e* alleles and mediate a partial CAP function leading to variable mild, dosage-sensitive effects. A mild, dosage-sensitive antimorphic character would also explain why the rather subtle morphological phenotypes are not enhanced in hemizygous embryos with the *CAP*-deleting deficiency *Df(2L)BSC281*. Specific antisera are required to detect such putative truncated protein isoforms. Unfortunately, all available CAP antisera are directed to the conserved CAP C-terminus that is affected by the *CAP* excision mutants.

Currently, we can only speculate what causes the observed increase of CB numbers in *CAP* mutants. CB number has been shown to be positively and negatively regulated by the antagonistic actions of receptor tyrosine kinase (RTK/MAPK) and Notch signaling, respectively [69–73]. It is conceivable that CAP could influence signaling efficiency either through its proposed impact on cell adhesion or by interfering with the activity or distribution of certain signaling components (e.g. via its SH3 domains). Interestingly, the vertebrate CAP-homolog CAP/Ponsin negatively regulates MAPK signaling [44] by binding to components of the MAPK-pathway [74]. Alternatively, Integrin dependent adhesion, mediated by cell-cell or cell-extracellular matrix contacts, could serve as a mechanism to control cell division as has been reported in many mammalian cell types (reviewed in [75]). Changing the stability of the IAC by *CAP* mutation may interfere with such controls and trigger signals to initiate unscheduled cell division. This aspect of the cardiac CAP phenotype needs further investigation, although such investigations may be complicated by the subtleness of the phenotype and the complexity of CB number-determining signaling processes.

In the process of tube formation the CBs change their cell shape and cell surface properties. This requires a dramatic reorganization of their ECM, adhesion molecules and cytoskeletal organization [17]. CAP plays an important role in these processes since *CAP* mutants result in strong defects in lumen formation. CAP is most prominent at the luminal periphery of CBs where it colocalizes with ßPS1-Integrin, Talin and Slit, which are all essential for lumen formation. Integrin is the crucial player since it counteracts luminal spreading of Discs-large to the luminal domain and supports luminal location of Robo/Slit and the formation of a non-adhesive luminal ECM [18]. In *CAP* mutants the polarized Integrin distribution is maintained but the luminal domain of CBs stained for Integrin looks ill-defined and more expanded than in controls. This is similar to the aberrant broad distribution of ßPS1-Integrin at muscle attachment sites of somatic muscles in *CAP* mutant animals [51]. Interestingly, Talin is affected in a similar way. In contrast, CAP localization is not dramatically changed in zygotic *rhea*^*79*^ mutations; the observed changes were interpreted as secondary to the general perturbations in heart morphology by the *rhea*^*79*^ mutation [38]. In summary, this suggests a role for CAP in fine-tuning integrin-dependent adhesion that does not affect CB polarization and is upstream or independent of Talin-mediated functions at the luminal domain. Slit localization, though perturbed, is still observed at its original domains and an apparently normal basal membrane lining is still formed between contralateral CBs. Therefore, the adhesive properties of the luminal ECM might be unchanged, suggesting, that *CAP* mutants fail to establish a lumen not because of changes in adhesiveness, but owing to defects in the cytoskeletal architecture relevant for the formation of crescent shaped CBs. Due to defective Integrin assembly or its coupling to the cytoskeleton, *CAP* mutant CBs may be impaired in forming the functional connections between focal adhesions and the actin cytoskeleton that are required for the establishment of crescent cell shape. This is consistent with the observed role of CAP for the induction of stress fibers and focal adhesions in various mammalian cell types, and fits well with the proposed role for CAP and other mammalian SORBS family proteins in stiffness-sensing and force transmission [47, 76].

In the transition of the late embryo to the 1^st^ instar larva and during pupation the dorsal vessel undergoes terminal differentiation to form the contractile larval and adult heart, respectively. This includes the establishment of robust links between the cytoskeleton and myofibrils and the cell contacts between contralateral CMCs. These contacts are structurally and functionally similar to intercalated discs in the vertebrate heart, which sense and transmit tension across the heart muscle [77]. Integrin and Talin play an important role in establishment and lifelong maintenance of CMC adhesion and their loss of function results in a severe retraction of myofibrils, losses of contacts between ipsilateral and contralateral myocytes and severely impaired function of the adult heart [6, 7]. CAP staining closely correlates with the Integrin localization at contact sites of contralateral CMCs and near Z-bands. Integrin is thought to contribute to the costamere structures that link the Z-bands to the ECM (reviewed in [35]), supported by proteins like ZASP that bind to and organize Z-bands and their Integrin connection [78]. The strong CAP-ßPS1-Integrin colocalization near Z-bands and in circular myofibrils suggests association of CAP with costameres, consistent with earlier observations made by Zhang and coworkers for the CAP homolog in mouse CMCs [48]. However, this has to be established by using more protein marker combinations at higher resolution.

Our physiological tests of *CAP* mutant larvae and adults uncovered animals with a reduced heart function. Apparent fractional shortening in larvae and pumping efficiency in adults were strongly affected in *CAP* mutants, although both parameters showed a similar incomplete penetrance as was observed for morphological defects in embryos. The physiological defects in heart function correlate with a locally defective morphology of adult *CAP* mutant hearts. A similar phenotype was found following Talin RNAi, when the knock down was limited to 2^nd^ larval stage [7]. Also, *hand*-GAL4 driven knock down of Integrin-linked kinase ILK showed a similar phenotype [6]. Our data indicate that CAP is required for the maintenance and correct assembly of IACs at contact sites of contralateral CMCs and their connections to myofibrils. This function is in correspondence with its suggested role of CAP at muscle attachment sites of somatic muscles [51].

Overexpression of IAC components in *Drosophila* hearts results in premature aging and the age dependent increase in cardiac arrhythmias that could be prevented by reducing the gene dosage in heterozygous *ilk* and *mys* animals or by moderate cardiac specific knockdown of other IAC components [6]. Elimination of *Talin1*, one of two *Talin* genes in mice, also reduced the hypertrophic responses to increased cardiac load [79]. However, strong downregulation of Talin and other major components of the IAC cause serious defects, similar to dilated cardiomyopathy [6, 7]. Compared to other IAC proteins CAP depletion has rather mild effects on *Drosophila* heart structure and function. This and the incomplete penetrance of the phenotype classify CAP as a conserved modifier of IAC function that may provide a useful tool in the therapies of age dependent heart diseases.

## Supporting information

S1 Fig Expression of *CAP* transcripts as detected by *in situ* hybridization. Shown are lateral (a, c) or dorsal (b, d, e, f) views; anterior left. (a) Stage 11: first *CAP* expression in Garland cells (arrow). (b) Stage 13/14: *CAP* expression in the rows of cardioblasts (arrows; lower row out of focus). (c) Stage 14: cardioblasts (arrow) migrating dorsally. (d) Stage 15: the two rows of cardioblasts are approaching the dorsal midline (arrow). (e) Stage 16: the dorsally aligned cardioblast rows form the DV with a distinct posterior heart tube (arrow). (f) Stage 16 embryo hybridized with the 5´*CAP* sense probe: no signal; bar: 35 μm.
(PDF)

S2 Fig Colocalization of CAP with Kettin, ZASP and Integrin in the adult heart tube. Colocalization of CAP using CAP antiserum combined with either Kettin-GFP expression, ZASP52-GFP expression or ßPS1-Integrin antibodies (a-f): Optical sections stained with CAP antiserum (a, d) with Kettin-GFP expression (b, e); merge (c, f). Focus to ventral longitudinal muscles (a-c) or to circular myofibrils (d-f). Bars: 5 μm. (g-l) Optical sections stained with CAP antiserum (g, j) with ZASP52-GFP expression (h, k); merge (i, l). Focus to circular myofibrils (g-i); heart at lower magnification focusing to ventral longitudinal muscles, to contact sites of contralateral CMCs (open arrows) and pericardial cells (j-l). Bars in (g-i): 5 μm, (j-l): 10 μm. (m-r) Optical sections stained with CAP antiserum (m, p), ßPS1-Integrin antibodies (n, q); merge (o, r). Focus to myofibrillar region and contact sites of contralateral CMCs (arrows; m-o); heart at lower magnification focusing to contact sites of contralateral CMCs (open arrows) and pericardial cells (p-r). Bars in (m-o): 6 μm, (p-r): 15 μm.
(PDF)

S3 Fig Phenotype of *CAP* RNAi. (a) Embryo with *UAS-CAP RNAi* driven by *tinC+D-GAL4*: One segment is disrupted by the loss of two Mef2^+^ CBs in the lower hemisegment (white arrows). Pairing with contralateral cells is delayed in the middle region. (b, c) Embryo with *UAS-CAP* RNAi driven by *twi+how-GAL4* as inducer. (b) Note a group of 3 ostial cells in the heart region (3 arrows); (c) two central CBs in the posterior aorta (arrows). Bars: 10 μm.
(TIF)

S4 Fig Entire uncropped Western blot used for Fig 1b. Uncropped Western blot using adult whole fly extracts reacted with either rabbit anti-CAP (top) or anti-H2A (loading control below; see also Fig 1b). The type of extracts loaded is as follows: Lane 1: CAP-GFP, Lane 2: CAP RNAi (*w*^*1118*^;* UAS-CAP RNAi; da-GAL4)*, Lane 3: white RNAi (*w*^*1118*^;* UAS-white RNAi; da-GAL4)*, Lane 4: *CAP*^*42b*^ [51], Lane 5: *CAP*^*49e*^ [51], Lane 6: RNAi-control (*w*^*1118*^), Lane 7: protein molecular weight marker, Lane 8: *CAP*^*42b*^;* hand-GFP*, Lane 9: *CAP*^*49e*^;* hand-GFP*. The single 43 kD MW^app^ peptide stained in CAP mutant extracts (lanes 4 and 5; omitted in Fig 1b) is probably an artifact. It is not detected in *CAP; hand-GFP* mutant extracts (lanes 8, 9) or following RNAi (lane 2, 3). It is not or only marginally (overflow of adjacent lane?) present in control extracts (lanes 1, 6) and was not detected by Bharadwaj and coworkers (Fig 1D in [51]).
(DOCX)

S1 Movie Live imaging of normal heart beat in *w*^*1118*^ control L3 larvae carrying the *handC3*.*1-GFP* reporter. Imaging was performed as described in materials and methods.
(RAR)

S2 Movie Live imaging of aberrant heart beat in *CAP*^*49e*^ L3 larvae carrying the *handC3*.*1-GFP* reporter. (RAR)

S1 File Evaluation and statistics. 1. Evaluation of cardioblast numbers in DVs of different genotype: top line indicates the genotype of the embryos evaluated; second line lists the type of CBs evaluated and the third and following lines list the numbers of cells counted per embryo (compare Table 1). Line 32 displays the average for the CB numbers of the given genotype, followed by corresponding the standard deviation (S) in line 33 and the variance (S) in line 34. Line 35 lists the p-scores obtained by a 2-sided t-test with equal variance (type 2) in relation to *w1118*; line 36 the same in relation to *CAP*^*42b*^, line 37 in relation to *CAP*^*49e*^ and line 38 in relation to CAP RNAi control without GAL4-driver (compare Table 1). 2. Evaluation of heart contraction cycle by live imaging. 2.1. lists the maximal (max) and minimal (min) distance of CMC nuclei close to the inner valve cells for *hand-GFP;w*^*1118*^ controls measured for each 10 cycles of heart beat per animal. The length determined for DCN^max^ (max) and DCN^min^ (min) are listed next to the avi file name and time point of the records in sec. Average and standard deviation for DCN^max^ and DCN^min^ were calculated. 2.2. Same is displayed for *hand-GFP; CAP49e*; 2.3. Evaluation of the data. Listed to the left are the values of AFS[%] calculated from the average DCN^max^ and DCN^min^ for each *hand-GFP;CAP*^*49e*^ and *hand-GFP; w*^*1118*^ larva according to: [(DCN^max^–DCN^min^)/DCN^max^] x 100%. From this the average and standard deviation for the whole group was determined for each genotype, listed in the table and used to calculate the variance. Then the p-scores were calculated using a 2-sided t-test with unequal variance (type 3). 3. Evaluation of heart transport activity of pharate adults determined by dye angiography. **3.1** Transport rate as average pixel intensity in ROI (*w*^*1118*^) lists the pixel values determined for three *w*^*1118*^ adults at different time points (for details see Materials and methods section) along with their average and standard deviation. **3.2** shows the same for 7 homozygous *CAP*^*42b*^ animals and **3.3** the same for 6 *CAP*^*49e*^ animals. These single fly values were graphically displayed in Fig 8e, 8g and 8i. **3.4** lists the average and the standard deviation of pixel intensities for each genotype that were graphically displayed in Fig 8d, 8f and 8h. From these data the p-scores to *w*^*1118*^ and significance were evaluated by a 2-sided t-test with unequal variance.
(PDF)
