# Supplementary figures and images for: Cbl-Associated Protein CAP contributes to correct formation and robust function of the Drosophila heart tube
Source: PLoS One. 2020 May 29;15(5):e0233719. doi: 10.1371/journal.pone.0233719 (PMC7259718; doi:10.1371/journal.pone.0233719)

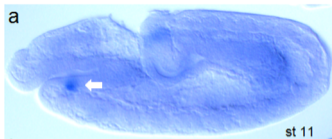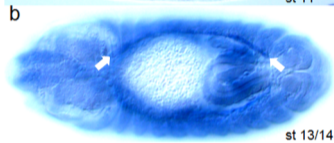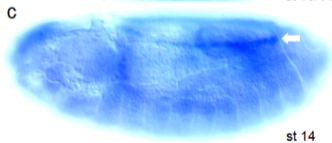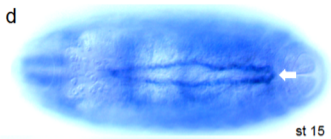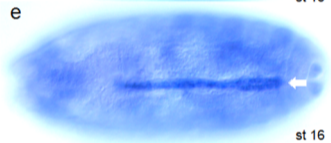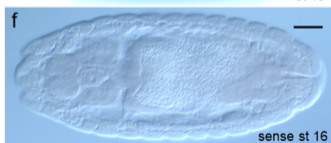

Supplement: S1 Fig — Shown are lateral (a, c) or dorsal (b, d, e, f) views; anterior left. (a) Stage 11: first CAP expression in Garland cells (arrow). (b) Stage 13/14: CAP expression in the rows of cardioblasts (arrows; lower row out of focus). (c) Stage 14: cardioblasts (arrow) migrating dorsally. (d) Stage 15: the two rows of cardioblasts are approaching the dorsal midline (arrow). (e) Stage 16: the dorsally aligned cardioblast rows form the DV with a distinct posterior heart tube (arrow). (f) Stage 16 embryo hybridized with the 5´CAP sense probe: no signal; bar: 35 μm. (PDF) [file pone.0233719.s001.pdf]

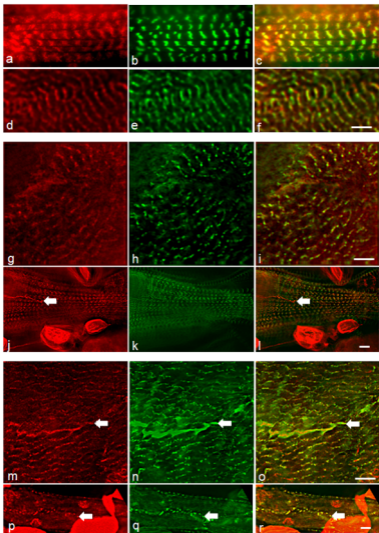

Supplement: S2 Fig — Colocalization of CAP using CAP antiserum combined with either Kettin-GFP expression, ZASP52-GFP expression or ßPS1-Integrin antibodies (a-f): Optical sections stained with CAP antiserum (a, d) with Kettin-GFP expression (b, e); merge (c, f). Focus to ventral longitudinal muscles (a-c) or to circular myofibrils (d-f). Bars: 5 μm. (g-l) Optical sections stained with CAP antiserum (g, j) with ZASP52-GFP expression (h, k); merge (i, l). Focus to circular myofibrils (g-i); heart at lower magnification focusing to ventral longitudinal muscles, to contact sites of contralateral CMCs (open arrows) and pericardial cells (j-l). Bars in (g-i): 5 μm, (j-l): 10 μm. (m-r) Optical sections stained with CAP antiserum (m, p), ßPS1-Integrin antibodies (n, q); merge (o, r). Focus to myofibrillar region and contact sites of contralateral CMCs (arrows; m-o); heart at lower magnification focusing to contact sites of contralateral CMCs (open arrows) and pericardial cells (p-r). Bars in (m-o): 6 μm, (p-r): 15 μm. (PDF) [file pone.0233719.s002.pdf]

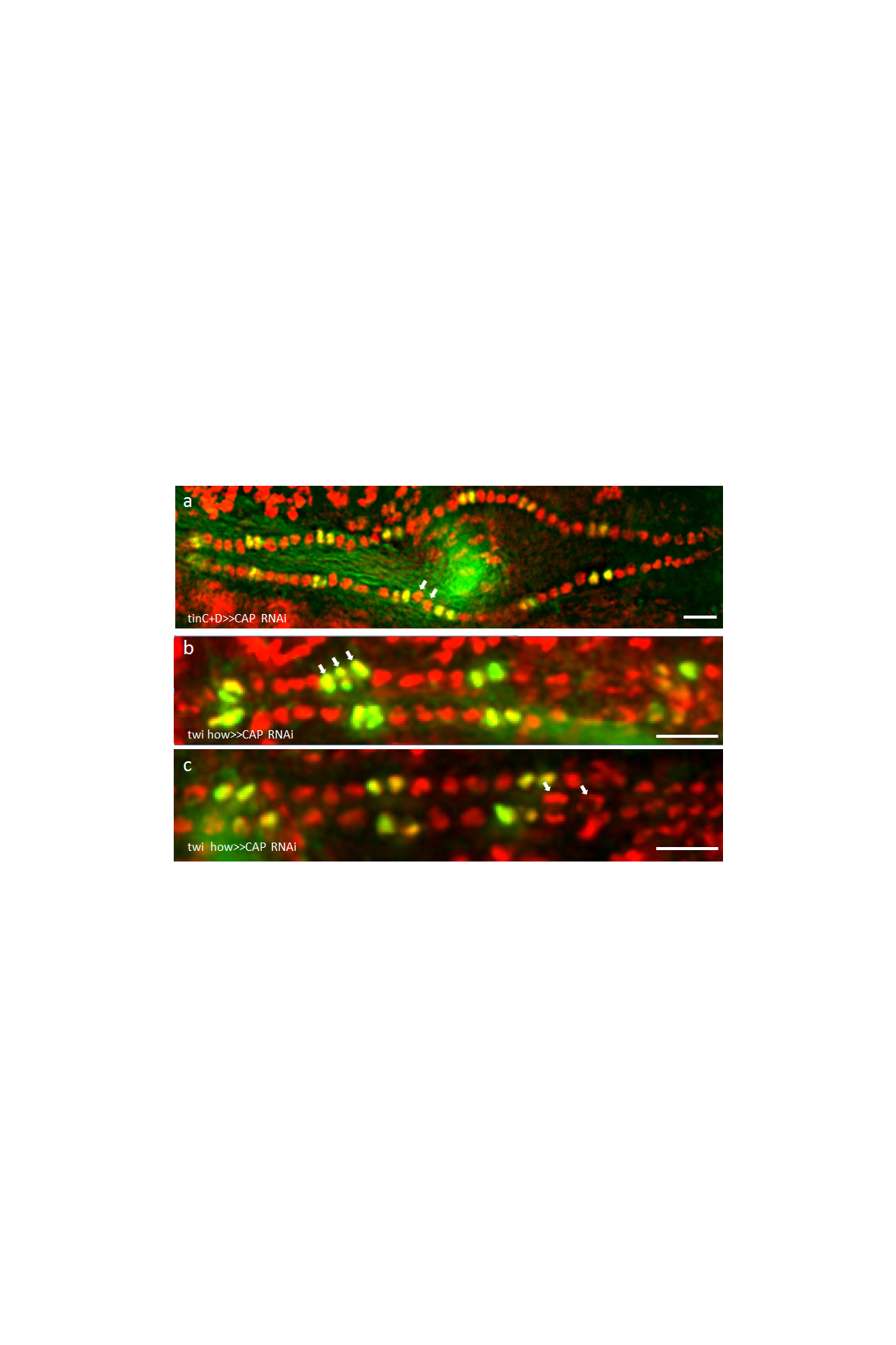

Supplement: S3 Fig — (a) Embryo with UAS-CAP RNAi driven by tinC+D-GAL4: One segment is disrupted by the loss of two Mef2+ CBs in the lower hemisegment (white arrows). Pairing with contralateral cells is delayed in the middle region. (b, c) Embryo with UAS-CAP RNAi driven by twi+how-GAL4 as inducer. (b) Note a group of 3 ostial cells in the heart region (3 arrows); (c) two central CBs in the posterior aorta (arrows). Bars: 10 μm. (TIF) [file pone.0233719.s003.tif]
