# Supplementary material for: Cbl-Associated Protein CAP contributes to correct formation and robust function of the Drosophila heart tube
Source: PLoS One. 2020 May 29;15(5):e0233719. doi: 10.1371/journal.pone.0233719 (PMC7259718; doi:10.1371/journal.pone.0233719)
Supplement: S4 Fig — Uncropped Western blot using adult whole fly extracts reacted with either rabbit anti-CAP (top) or anti-H2A (loading control below; see also Fig 1b). The type of extracts loaded is as follows: Lane 1: CAP-GFP, Lane 2: CAP RNAi (w1118; UAS-CAP RNAi; da-GAL4), Lane 3: white RNAi (w1118; UAS-white RNAi; da-GAL4), Lane 4: CAP42b [51], Lane 5: CAP49e [51], Lane 6: RNAi-control (w1118), Lane 7: protein molecular weight marker, Lane 8: CAP42b; hand-GFP, Lane 9: CAP49e; hand-GFP. The single 43 kD MWapp peptide stained in CAP mutant extracts (lanes 4 and 5; omitted in Fig 1b) is probably an artifact. It is not detected in CAP; hand-GFP mutant extracts (lanes 8, 9) or following RNAi (lane 2, 3). It is not or only marginally (overflow of adjacent lane?) present in control extracts (lanes 1, 6) and was not detected by Bharadwaj and coworkers (Fig 1D in [51]). (DOCX) [file pone.0233719.s004.docx]

**S7 Fig : Uncropped Western Blot data of the blot shown in Fig 1b**

Lane 1 2 3 4 5 6 7 8 9


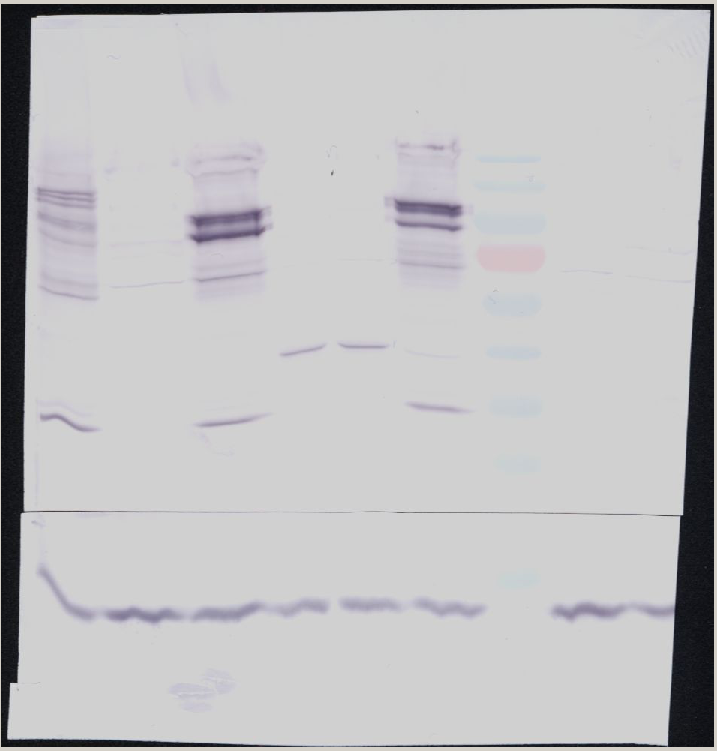


**Entire Western blot using adult adult fly extracts** reacted with either rabbit anti-CAP (top) or anti-H2A (loading control below; see also Fig 1b). The type of extracts loaded is as follows: Lane 1: CAP-GFP, Lane 2: CAP RNAi (*w^1118^; UAS-CAP* *RNAi ; da-GAL4),* Lane 3: white RNAi (*w^1118^; UAS-white RNAi; da-GAL4)*, Lane 4: *CAP^42b^* [51] , Lane 5: *CAP^49e^* [51], Lane 6: RNAi-control (*w^1118^*), Lane 7: protein molecular weight marker, Lane 8: *CAP^42b^; hand-GFP*, Lane 9: *CAP^49e^; hand-GFP*. The single 43 kD MW^app^ peptide stained in CAP mutant extracts lanes 4 and 5 (omitted in Fig 1b) is probably an artifact. It is not detected in *CAP* mutant*; hand-GFP* extracts (lanes 8, 9) or following RNAi (lane 2, 3). It is not or only very weakly present in control extracts (lanes 1, 6) and was not detected by Bharadwaj and coworkers (Fig 1D in [51]).
