# Supplementary material for: Cbl-Associated Protein CAP contributes to correct formation and robust function of the Drosophila heart tube
Source: PLoS One. 2020 May 29;15(5):e0233719. doi: 10.1371/journal.pone.0233719 (PMC7259718; doi:10.1371/journal.pone.0233719)
Supplement: S1 File — 1. Evaluation of cardioblast numbers in DVs of different genotype: top line indicates the genotype of the embryos evaluated; second line lists the type of CBs evaluated and the third and following lines list the numbers of cells counted per embryo (compare Table 1). Line 32 displays the average for the CB numbers of the given genotype, followed by corresponding the standard deviation (S) in line 33 and the variance (S) in line 34. Line 35 lists the p-scores obtained by a 2-sided t-test with equal variance (type 2) in relation to w1118; line 36 the same in relation to CAP42b, line 37 in relation to CAP49e and line 38 in relation to CAP RNAi control without GAL4-driver (compare Table 1). 2. Evaluation of heart contraction cycle by live imaging. 2.1. lists the maximal (max) and minimal (min) distance of CMC nuclei close to the inner valve cells for hand-GFP;w1118 controls measured for each 10 cycles of heart beat per animal. The length determined for DCNmax (max) and DCNmin (min) are listed next to the avi file name and time point of the records in sec. Average and standard deviation for DCNmax and DCNmin were calculated. 2.2. Same is displayed for hand-GFP; CAP49e; 2.3. Evaluation of the data. Listed to the left are the values of AFS[%] calculated from the average DCNmax and DCNmin for each hand-GFP;CAP49e and hand-GFP; w1118 larva according to: [(DCNmax–DCNmin)/DCNmax] x 100%. From this the average and standard deviation for the whole group was determined for each genotype, listed in the table and used to calculate the variance. Then the p-scores were calculated using a 2-sided t-test with unequal variance (type 3). 3. Evaluation of heart transport activity of pharate adults determined by dye angiography. 3.1 Transport rate as average pixel intensity in ROI (w1118) lists the pixel values determined for three w1118 adults at different time points (for details see Materials and methods section) along with their average and standard deviation. 3.2 shows the same for 7 h [file pone.0233719.s007.pdf]

1. Evaluation of cardioblast numbers in DVs of different genotype

	w1118		*CAP42b*		*CAP49e*		*CAP42b/Df(2R)BSC281*		*CAP49e/Df(2R)BSC281*	
	Mef+	Doc+	Mef+	Doc+	Mef+	Doc+	Mef+	Doc+	Mef+	Doc+
	104	28	106	28	105	28	106	28	106	32
	104	28	109	28	104	29	106	28	112	28
	104	28	106	28	106	28	104	28	104	28
	104	28	112	29	102	29	109	31	108	28
	104	28	106	30	104	28	106	28	104	29
	103	28	109	32	110	29	108	28	104	30
	104	28	106	29	106	30	104	28	104	28
	104	28	106	28	112	29	104	27	104	28
	103	28	108	28	106	28	108	28		
	104	28	112	29	104	28	108	30		
	104	28	104	28	107	28				
	104	28	110	29	108	28				
	104	28	112	29	104	28				
	104	28	108	28	108	28				
	104	28	106	28	107	29				
	102	29	106	28	108	28				
	100	30	104	28	114	28				
	100	30	108	28	106	29				
	104	27	108	28	104	28				
	104	28	108	29	107	29				
	104	28	104	28	107	29				
	104	28	106	28	109	32				
			110	28	106	29				
			106	28	104	28				
			104	28	106	28				
			110	28	105	29				
			108	28	104	28				
					104	28				
					104	28				
Average	103,45	28,18	107,48	28,44	106,24	28,55	106,30	28,40	105,75	28,88
Standard deviation	1,22	0,66	2,44	0,89	2,63	0,87	1,89	1,17	2,92	1,46
Variance	1,50	0,44	6,07	0,93	7,92	0,91	3,57	1,38	8,50	2,13
t-Test (2,2) (to w1118)	1	1	7,0036E-09	0,25773047	2,98074E-05	0,10334231	1,6323E-05	0,50605643	0,00454911	0,08137021
t-Test (2,2) (to CAP42b)							0,17592471	0,90240195		
t-Test (2,2) (to CAP49e)									0,64991005	0,43045122
t-Test (2,2) (to CAPRNAi control)										

Rhea1/CAP		Scb/CAP		tinD;tinc/CAPRNAi		twist;how x CAPRNAi		CAPRNAi control		CAP49e rescue by CAP-PC		
Mef+	Doc+	Mef+	Doc+	Mef+	Doc+	Mef+	Doc+	Mef+	Doc+	Mef+	Doc+	
104	28	104	28	104	28	104	28	104	28	104	29	
108	29	104	28	104	28	104	28	104	29	104	29	
108	28	104	28	104	28	104	28	104	28	104	29	
106	28	104	28	102	28	106	28	104	28	104	27	
104	28	104	29	104	28	106	28	104	28	104	28	
104	28	104	28	104	28	104	29	104	28	104	28	
		106	28	102	27	103	30	104	28	104	28	
		102	28	104	28	104	29	105	29	106	28	
		104	28	105	28	104	28					
		104	28	104	28	104	29					
		106	28	104	28	104	29					
				104	28	104	29					
						104	29					
						102	29					
105,67	28,17	104,18	28,09	103,75	27,92	104,07	28,64	104,13	28,25	104,25	28,25	Average
1,97	0,41	1,08	0,30	0,87	0,29	1,00	0,63	0,35	0,46	0,71	0,71	Standard deviation
3,87	0,17	1,16	0,09	0,75	0,08	0,99	0,40	0,13	0,21	0,50	0,50	Variance
0,00199213	0,95832115	0,10489635	0,6704851	0,46522861	0,1997381	0,1235329	0,04652432	0,14192258	0,79200184	0,09566055	0,80861672	t-Test (2,2) (to w1118)
												t-Test (2,2) (to CAP42b)
										0,0427401	0,37433768	t-Test (2,2) (to CAP49e)
				0,26362905	0,06163422	0,88577588	0,14171879					t-Test (2,2) (to CAPRNAi control)

2. Evaluation of heart contraction cycle by life imaging

2.1. Maxima and minima of 10 cycles (*HandGFP;w*^{1118})

	Label	Length		Label	Length		Label	Length
max	HandGFPKO10x01_R3D.avi: 2.57s	144,84	max	HandGFPKO10x02_R3D.avi: 15.14s	98,42	max	HandGFPKO10x03_R3D.avi: 13.71s	96,25
	HandGFPKO10x01_R3D.avi: 4.57s	135,77		HandGFPKO10x02_R3D.avi: 15.71s	88,81		HandGFPKO10x03_R3D.avi: 14,29s	108,63
	HandGFPKO10x01_R3D.avi: 6.00s	136,68		HandGFPKO10x02_R3D.avi: 16.43s	87,48		HandGFPKO10x03_R3D.avi: 15.71s	95,27
	HandGFPKO10x01_R3D.avi: 7.29s	121,26		HandGFPKO10x02_R3D.avi: 16.86s	92,18		HandGFPKO10x03_R3D.avi: 16.43s	97,08
	HandGFPKO10x01_R3D.avi: 8.57s	132,62		HandGFPKO10x02_R3D.avi: 17,43s	94,54		HandGFPKO10x03_R3D.avi: 16.86	81,44
	HandGFPKO10x01_R3D.avi: 20.43s	124,79		HandGFPKO10x02_R3D.avi: 18.00s	137,62		HandGFPKO10x03_R3D.avi: 17.29s	97.14
	HandGFPKO10x01_R3D.avi: 31.00s	130,97		HandGFPKO10x02_R3D.avi: 18.57s	121,26		HandGFPKO10x03_R3D.avi: 17.57s	95,85
	HandGFPKO10x01_R3D.avi: 32.29s	121,31		HandGFPKO10x02_R3D.avi: 19,29	124,06		HandGFPKO10x03_R3D.avi: 21.57s	97,99
	HandGFPKO10x01_R3D.avi: 33.29s	110,72		HandGFPKO10x02_R3D.avi: 21,00s	89,02		HandGFPKO10x03_R3D.avi: 22.71s	80,62
	HandGFPKO10x01_R3D.avi: 35,29s	136,86		HandGFPKO10x02_R3D.avi: 22,14s	94,02		HandGFPKO10x03_R3D.avi: 23.00s	85,09
	Average	129,58			102,74			93,14
	Standard deviation	9,50			17,04			8,56
	Max	144,84			137,62			108,63
	Min	110,72						
min	HandGFPKO10x01_R3D.avi: 3.14s	53,41	min	HandGFPKO10x02_R3D.avi: 15.57s	49,24	min	HandGFPKO10x03_R3D.avi: 14.00s	63,25
	HandGFPKO10x01_R3D.avi: 5.14s	61,29		HandGFPKO10x02_R3D.avi: 16.14	48,17		HandGFPKO10x03_R3D.avi: 14.86s	58,87
	HandGFPKO10x01_R3D.avi: 6.86s	58,26		HandGFPKO10x02_R3D.avi: 16.71s	47,85		HandGFPKO10x03_R3D.avi: 15,86	53,49
	HandGFPKO10x01_R3D.avi: 7.57s	78.26		HandGFPKO10x02_R3D.avi: 17.14s	42,95		HandGFPKO10x03_R3D.avi: 16,57s	50,61
	HandGFPKO10x01_R3D.avi: 9.00s	60,44		HandGFPKO10x02_R3D.avi: 17,71s	59,08		HandGFPKO10x03_R3D.avi: 17.00s	67,05
	HandGFPKO10x01_R3D.avi: 20.00s	60,61		HandGFPKO10x02_R3D.avi: 18,29	85,62		HandGFPKO10x03_R3D.avi: 17.43s	48,76
	HandGFPKO10x01_R3D.avi: 30.43s	60.11		HandGFPKO10x02_R3D.avi: 18.86s	74,81		HandGFPKO10x03_R3D.avi: 17.86s	50,33
	HandGFPKO10x01_R3D.avi: 31.57s	54,71		HandGFPKO10x02_R3D.avi: 20.00s	35,01		HandGFPKO10x03_R3D.avi: 21.43s	60,54
	HandGFPKO10x01_R3D.avi: 32.71s	58,52		HandGFPKO10x02_R3D.avi: 21,43	30,59		HandGFPKO10x03_R3D.avi: 22.00s	58,82
	HandGFPKO10x01_R3D.avi: 33.71s	63,82		HandGFPKO10x02_R3D.avi: 22,71	28,86		HandGFPKO10x03_R3D.avi: 22.86s	54,59
	Mean	58,88			50,22			56,63
	STABW.N	3,23			17,53			5,74
	Max	63,82			85,62			67,05
	Min	53,41			28,86			48,76

2.2. Maxima and minima of consecutive cycles (*CAP*^{49e})

Evaluation Life Imaging CAP49e

	Label	Length		Label	Length		Label	Length
max	CAP49eHandGFP10x02_R3D.avi: 0.71s	127,25	max	CAP49eHandGFP10x03_R3D.avi: 5.86s	190,69	max	CAP49eHandGFP10x04_R3D.avi: 5.00s	128,55
	CAP49eHandGFP10x02_R3D.avi: 1.43s	125,06		CAP49eHandGFP10x03_R3D.avi: 6.43s	192,72		CAP49eHandGFP10x04_R3D.avi: 7.00s	109,88
	CAP49eHandGFP10x02_R3D.avi: 1.71s	129,14		CAP49eHandGFP10x03_R3D.avi: 7.14s	192.35		CAP49eHandGFP10x04_R3D.avi: 9.00s	44,00
	CAP49eHandGFP10x02_R3D.avi: 2.14s	109,00		CAP49eHandGFP10x03_R3D.avi: 7.86s	177,41		CAP49eHandGFP10x04_R3D.avi: 11.00s	46,32
	CAP49eHandGFP10x02_R3D.avi: 2.43s	118,21		CAP49eHandGFP10x03_R3D.avi: 8.57s	190,42		CAP49eHandGFP10x04_R3D.avi: 13.00s	75,47
	CAP49eHandGFP10x02_R3D.avi: 3.14s	113,00		CAP49eHandGFP10x03_R3D.avi: 9.29s	183,44		CAP49eHandGFP10x04_R3D.avi: 15.00s	133,65
	CAP49eHandGFP10x02_R3D.avi: 4.71	118,00		CAP49eHandGFP10x03_R3D.avi: 11.57s	190,34		CAP49eHandGFP10x04_R3D.avi: 17.00s	140,07
	CAP49eHandGFP10x02_R3D.avi: 5.43	118,02		CAP49eHandGFP10x03_R3D.avi: 12.43s	172,53		CAP49eHandGFP10x04_R3D.avi: 19.00s	141,11
	CAP49eHandGFP10x02_R3D.avi: 6.14s	109,29		CAP49eHandGFP10x03_R3D.avi: 13.14s	182,24		CAP49eHandGFP10x04_R3D.avi: 21.00s	65,76
	CAP49eHandGFP10x02_R3D.avi:	107,00		CAP49eHandGFP10x03_R3D.avi: 14.00s	175,91		CAP49eHandGFP10x04_R3D.avi: 23.00s	44,27
	Average	117,40			183,97			92,91
	Standard deviation	7,88			7,04			39,67
	MAX	129,14			192,72			141,11
	MIN	107,00			172,53			44,00
min	CAP49eHandGFP10x02_R3D.avi: 1.14s	96.02	min	CAP49eHandGFP10x03_R3D.avi: 5.43s	133,81	min	CAP49eHandGFP10x04_R3D.avi: 6.00s	136,78
	CAP49eHandGFP10x02_R3D.avi: 1.57s	118,07		CAP49eHandGFP10x03_R3D.avi: 6.14s	133,24		CAP49eHandGFP10x04_R3D.avi: 8.00s	45,04
	CAP49eHandGFP10x02_R3D.avi: 1.86s	101,27		CAP49eHandGFP10x03_R3D.avi: 6.86s	131,94		CAP49eHandGFP10x04_R3D.avi: 10.00s	41.11
	CAP49eHandGFP10x02_R3D.avi: 2.29s	94,89		CAP49eHandGFP10x03_R3D.avi: 7.57s	127,09		CAP49eHandGFP10x04_R3D.avi: 12.00s	45,61
	CAP49eHandGFP10x02_R3D.avi: 2.71s	93,02		CAP49eHandGFP10x03_R3D.avi: 8.29s	125,42		CAP49eHandGFP10x04_R3D.avi: 14.00s	116,39
	CAP49eHandGFP10x02_R3D.avi: 3.57s	105,30		CAP49eHandGFP10x03_R3D.avi: 9.00s	136,56		CAP49eHandGFP10x04_R3D.avi: 16.00s	141,45
	CAP49eHandGFP10x02_R3D.avi: 5.00s	95,89		CAP49eHandGFP10x03_R3D.avi: 9,71s	123,69		CAP49eHandGFP10x04_R3D.avi: 18.00s	137,00
	CAP49eHandGFP10x02_R3D.avi: 5.71	88,14		CAP49eHandGFP10x03_R3D.avi: 11.86s	133,15		CAP49eHandGFP10x04_R3D.avi: 20.00s	142,90
	CAP49eHandGFP10x02_R3D.avi: 6.43s	91,14		CAP49eHandGFP10x03_R3D.avi: 12,71s	121,28		CAP49eHandGFP10x04_R3D.avi: 22.00s	46,32
	CAP49eHandGFP10x02_R3D.avi:	88,01		CAP49eHandGFP10x03_R3D.avi: 12.57s	120,60		CAP49eHandGFP10x04_R3D.avi: 24.00s	44,94
	Average	97,30			128,68			95,16
	Standard deviation	9,10			5,46			45,01
	MAX	118,07			136,56			142,90
	MIN	88,01			120,60			44,94

	Label	Length		Label	Length
max	CAP49eHandGFP10x05_R3D.avi: 5.00s	95,08	max	CAP49eHandGFP10x07_R3D.avi: 0.57s	136,67
	CAP49eHandGFP10x05_R3D.avi: 6.00s	96,15		CAP49eHandGFP10x07_R3D.avi: 1.00s	127,58
	CAP49eHandGFP10x05_R3D.avi: 7.00s	101,07		CAP49eHandGFP10x07_R3D.avi: 1.43s	110,22
	CAP49eHandGFP10x05_R3D.avi: 9.00s	98,41		CAP49eHandGFP10x07_R3D.avi: 1.86s	132,38
	CAP49eHandGFP10x05_R3D.avi: 11.00s	96,57		CAP49eHandGFP10x07_R3D.avi: 2.29s	137,30
	CAP49eHandGFP10x05_R3D.avi: 13.00s	99,93		CAP49eHandGFP10x07_R3D.avi: 2.71s	131,80
	CAP49eHandGFP10x05_R3D.avi: 17.00s	90,87		CAP49eHandGFP10x07_R3D.avi: 3.14s	133,96
	CAP49eHandGFP10x05_R3D.avi: 21.00s	96,61		CAP49eHandGFP10x07_R3D.avi: 3.57s	136,13
	CAP49eHandGFP10x05_R3D.avi: 25.00s	97,95		CAP49eHandGFP10x07_R3D.avi: 5.29s	98,86
	CAP49eHandGFP10x05_R3D.avi: 33.00s	93,91		CAP49eHandGFP10x07_R3D.avi: 5.71s	92,78
		96,66			123,77
		2,81			15,89
		101,07			137,30
		90,87			92,78
min	CAP49eHandGFP10x05_R3D.avi: 5.57s	94,81	min	CAP49eHandGFP10x07_R3D.avi: 0.71s	83,07
	CAP49eHandGFP10x05_R3D.avi: 6.57s	102,42		CAP49eHandGFP10x07_R3D.avi: 1.14s	63,13
	CAP49eHandGFP10x05_R3D.avi: 7.57s	98,30		CAP49eHandGFP10x07_R3D.avi: 1.57s	68,68
	CAP49eHandGFP10x05_R3D.avi: 9.57s	89,07		CAP49eHandGFP10x07_R3D.avi: 2.00s	72,56
	CAP49eHandGFP10x05_R3D.avi: 11.57s	98,68		CAP49eHandGFP10x07_R3D.avi: 2.43s	73,55
	CAP49eHandGFP10x05_R3D.avi: 13.57s	95,63		CAP49eHandGFP10x07_R3D.avi: 2.86s	72,33
	CAP49eHandGFP10x05_R3D.avi: 17.57s	99,28		CAP49eHandGFP10x07_R3D.avi: 3.29s	83,95
	CAP49eHandGFP10x05_R3D.avi: 21.57s	93,47		CAP49eHandGFP10x07_R3D.avi: 3.86s	71,59
	CAP49eHandGFP10x05_R3D.avi: 25.57s	97,13		CAP49eHandGFP10x07_R3D.avi: 5.43s	70,21
	CAP49eHandGFP10x05_R3D.avi: 33.57s	94,81		CAP49eHandGFP10x07_R3D.avi: 5.86s	69,03
		96,36			72,81
		3,49			6,03
		102,42			83,95
		89,07			63,13

max	CAP49eHandGFP10x08_R3D.avi: 0.86s	115,52	max	CAP49eHandGFP10x09_R3D.avi: 4.14s	148,27	max	CAP49eHandGFP10x12.avi: 2.86s	133,99
	CAP49eHandGFP10x08_R3D.avi: 1.86s	116,43		CAP49eHandGFP10x09_R3D.avi: 4.43s	147,35		CAP49eHandGFP10x12.avi: 3.86s	133,46
	CAP49eHandGFP10x08_R3D.avi: 2.86s	117,52		CAP49eHandGFP10x09_R3D.avi: 4.57s	144,92		CAP49eHandGFP10x12.avi: 4,86s	131,76
	CAP49eHandGFP10x08_R3D.avi: 3.86s	110,37		CAP49eHandGFP10x09_R3D.avi: 4.86s	154,73		CAP49eHandGFP10x12.avi: 5.86s	136,00
	CAP49eHandGFP10x08_R3D.avi: 4.57s	116,84		CAP49eHandGFP10x09_R3D.avi: 5.71s	160,18		CAP49eHandGFP10x12.avi: 6.86s	141,66
	CAP49eHandGFP10x08_R3D.avi: 5.57s	114,35		CAP49eHandGFP10x09_R3D.avi: 6.00s	164,83		CAP49eHandGFP10x12.avi: 8.86s	138,96
	CAP49eHandGFP10x08_R3D.avi: 6.29s	104,14		CAP49eHandGFP10x09_R3D.avi:6,29s	161,47		CAP49eHandGFP10x12.avi:10.86s	139,43
	CAP49eHandGFP10x08_R3D.avi: 7.29s	114,16		CAP49eHandGFP10x09_R3D.avi: 21.14s	159,39		CAP49eHandGFP10x12.avi: 14.86s	137,20
	CAP49eHandGFP10x08_R3D.avi: 8.14s	116,35		CAP49eHandGFP10x09_R3D.avi: 21.43s	165,34		CAP49eHandGFP10x12.avi: 18.86s	137,06
	CAP49eHandGFP10x08_R3D.avi: 9.14s	109,46		CAP49eHandGFP10x09_R3D.avi: 21.79	170,88		CAP49eHandGFP10x12.avi: 22.86s	134,63
	Average	113,51			157,74			136,42
	Standard deviation	4,04			8,21			2,89
	MAX	117,52			170,88			141,66
	MIN	104,14			144,92			131,76
min	CAP49eHandGFP10x08_R3D.avi: 1.43s	78,00	min	CAP49eHandGFP10x09_R3D.avi: 4.00s	136,31	min	CAP49eHandGFP10x12.avi: 3.14s	134,63
	CAP49eHandGFP10x08_R3D.avi: 2.29s	94,34		CAP49eHandGFP10x09_R3D.avi: 4.29s	136,93		CAP49eHandGFP10x12.avi: 4.14s	129,00
	CAP49eHandGFP10x08_R3D.avi: 3.43s	80,78		CAP49eHandGFP10x09_R3D.avi: 4.71s	143,56		CAP49eHandGFP10x12.avi: 5.14s	135,48
	CAP49eHandGFP10x08_R3D.avi: 4.29s	75,15		CAP49eHandGFP10x09_R3D.avi: 5.00s	144,51		CAP49eHandGFP10x12.avi: 6.14s	132,40
	CAP49eHandGFP10x08_R3D.avi: 5.00s	80,05		CAP49eHandGFP10x09_R3D.avi: 5.86s	142,37		CAP49eHandGFP10x12.avi: 7.14s	137,38
	CAP49eHandGFP10x08_R3D.avi: 6.00s	71,17		CAP49eHandGFP10x09_R3D.avi: 6.14s	135,06		CAP49eHandGFP10x12.avi: 9.14s	136,75
	CAP49eHandGFP10x08_R3D.avi: 6.71s	79,12		CAP49eHandGFP10x09_R3D.avi: 6.43s	151,29		CAP49eHandGFP10x12.avi:10.14s	138,76
	CAP49eHandGFP10x08_R3D.avi: 7.71s	69,86		CAP49eHandGFP10x09_R3D.avi: 21.29s	142,37		CAP49eHandGFP10x12.avi: 15.14s	137,91
	CAP49eHandGFP10x08_R3D.avi: 8.57s	73,74		CAP49eHandGFP10x09_R3D.avi: 21.57s	139,78		CAP49eHandGFP10x12.avi: 19.14s	136,57
	CAP49eHandGFP10x08_R3D.avi: 9.57s	74,73		CAP49eHandGFP10x09_R3D.avi: 21.86	152,86		CAP49eHandGFP10x12.avi:23.14s	137,38
	Average	77,69			142,50			135,63
	Standard deviation	6,55			5,67			2,80
	MAX	94,34			152,86			138,76
	MIN	69,86			135,06			129,00

max	CAP49eHandGFP10x13.avi: 8.14s	114,83	max	CAP49eHandGFP10x15.avi: 0.14s	66,85
	CAP49eHandGFP10x13.avi: 11.14s	132,85		CAP49eHandGFP10x15.avi: 1.29s	78,26
	CAP49eHandGFP10x13.avi: 12.43s	122,48		CAP49eHandGFP10x15.avi: 2.00s	70,72
	CAP49eHandGFP10x13.avi: 13.86s	146,73		CAP49eHandGFP10x15.avi: 2.71s	72,92
	CAP49eHandGFP10x13.avi: 15.14s	125,25		CAP49eHandGFP10x15.avi: 3.14s	74,09
	CAP49eHandGFP10x13.avi: 16.71s	112,61		CAP49eHandGFP10x15.avi: 4.29s	68,68
	CAP49eHandGFP10x13.avi: 18.29s	107,28		CAP49eHandGFP10x15.avi: 5.00s	64,56
	CAP49eHandGFP10x13.avi: 20,00s	118,26		CAP49eHandGFP10x15.avi: 5.43s	68,41
	CAP49eHandGFP10x13.avi: 31.14s	119,10		CAP49eHandGFP10x15.avi: 6.14s	72,11
	CAP49eHandGFP10x13.avi: 32.71s	103,75		CAP49eHandGFP10x15.avi: 6.86s	65,92
		120,31			70,25
		11,93			3,98
		146,73			78,26
		103,75			64,56
min	CAP49eHandGFP10x13.avi: 8.86s	50,25	min	CAP49eHandGFP10x15.avi: 0.29s	68,03
	CAP49eHandGFP10x13.avi: 11.86s	66,48		CAP49eHandGFP10x15.avi: 1.43s	72,95
	CAP49eHandGFP10x13.avi: 13.14s	49,40		CAP49eHandGFP10x15.avi: 2.29s	66,73
	CAP49eHandGFP10x13.avi: 14.57s	51,00		CAP49eHandGFP10x15.avi: 3.00s	72,45
	CAP49eHandGFP10x13.avi: 15.57s	66,85		CAP49eHandGFP10x15.avi: 3.29s	70,23
	CAP49eHandGFP10x13.avi: 17.43s	52,80		CAP49eHandGFP10x15.avi: 4.43s	64,54
	CAP49eHandGFP10x13.avi: 18.86s	72,95		CAP49eHandGFP10x15.avi: 5.14s	66,10
	CAP49eHandGFP10x13.avi: 20.86s	50,01		CAP49eHandGFP10x15.avi: 5.57s	64,78
	CAP49eHandGFP10x13.avi: 32.14s	41,59		CAP49eHandGFP10x15.avi: 6.29s	67,68
	CAP49eHandGFP10x13.avi: 33.14s	47,85		CAP49eHandGFP10x15.avi: 7.00s	68,01
		54,92			68,15
		9,62			2,77
		72,95			72,95
		41,59			64,54

max	CAP49eHandGFP10x16_R3D.avi: 2.57s	114,76	max	CAP49eHandGFP10x17.avi: 1.14s	98,41	max	CAP49eHandGFP10x18.avi: 0.57s	49,09
	CAP49eHandGFP10x16_R3D.avi: 3.29s	118,23		CAP49eHandGFP10x17.avi: 1.57s	97,08		CAP49eHandGFP10x18.avi: 1.57s	52,04
	CAP49eHandGFP10x16_R3D.avi: 4.00s	119,44		CAP49eHandGFP10x17.avi: 2.00s	99,39		CAP49eHandGFP10x18.avi: 2.57s	50,64
	CAP49eHandGFP10x16_R3D.avi: 4.71s	119,33		CAP49eHandGFP10x17.avi: 2.43s	97,05		CAP49eHandGFP10x18.avi: 3.57s	52,17
	CAP49eHandGFP10x16_R3D.avi: 10.43s	121,28		CAP49eHandGFP10x17.avi: 10.43s	104,22		CAP49eHandGFP10x18.avi: 4.57s	54,23
	CAP49eHandGFP10x16_R3D.avi: 10.86s	119,22		CAP49eHandGFP10x17.avi: 10.86s	110,02		CAP49eHandGFP10x18.avi: 9.57s	52,77
	CAP49eHandGFP10x16_R3D.avi: 11.43s	120,42		CAP49eHandGFP10x17.avi: 11.29s	106,45		CAP49eHandGFP10x18.avi: 14.57s	41,80
	CAP49eHandGFP10x16_R3D.avi: 11.86s	119,08		CAP49eHandGFP10x17.avi: 16.86s	110,46		CAP49eHandGFP10x18.avi: 19.57s	75,39
	CAP49eHandGFP10x16_R3D.avi: 12.86s	108,08		CAP49eHandGFP10x17.avi: 18.14s	94,92		CAP49eHandGFP10x18.avi: 24.57s	86,01
	CAP49eHandGFP10x16_R3D.avi: 13.14s	110,16		CAP49eHandGFP10x17.avi: 18.57s	89,89		CAP49eHandGFP10x18.avi: 29.57s	55,44
	Average	117,00			100,79			56,96
	Standard deviation	4,28			6,41			12,61
	MAX	121,28			110,46			86,01
	MIN	108,08			89,89			41,80
min	CAP49eHandGFP10x16_R3D.avi: 3.00s	94,43	min	CAP49eHandGFP10x17.avi: 1.29s	52,01	min	CAP49eHandGFP10x18.avi: 1.00s	44,41
	CAP49eHandGFP10x16_R3D.avi: 3.71s	85,91		CAP49eHandGFP10x17.avi: 1.71s	53,67		CAP49eHandGFP10x18.avi: 2.00s	54,08
	CAP49eHandGFP10x16_R3D.avi: 4.43s	85,42		CAP49eHandGFP10x17.avi: 2.14s	49,41		CAP49eHandGFP10x18.avi: 3.00s	49,04
	CAP49eHandGFP10x16_R3D.avi: 5.14s	89,89		CAP49eHandGFP10x17.avi: 2.71s	57,78		CAP49eHandGFP10x18.avi: 4.00s	62,65
	CAP49eHandGFP10x16_R3D.avi: 10.71s	97,53		CAP49eHandGFP10x17.avi: 10.71s	48,84		CAP49eHandGFP10x18.avi: 5.00s	44,18
	CAP49eHandGFP10x16_R3D.avi: 11.14s	88,26		CAP49eHandGFP10x17.avi: 11.14s	50,33		CAP49eHandGFP10x18.avi: 10.00s	47,04
	CAP49eHandGFP10x16_R3D.avi: 11.71s	103,81		CAP49eHandGFP10x17.avi: 11.57s	47,01		CAP49eHandGFP10x18.avi: 15.00s	47,04
	CAP49eHandGFP10x16_R3D.avi: 12.14s	82,35		CAP49eHandGFP10x17.avi: 17.14s	50,61		CAP49eHandGFP10x18.avi: 20.00s	70,66
	CAP49eHandGFP10x16_R3D.avi: 13.00s	90,38		CAP49eHandGFP10x17.avi: 18,43s	32,25		CAP49eHandGFP10x18.avi: 25.00s	85,38
	CAP49eHandGFP10x16_R3D.avi: 13.43s	93,47		CAP49eHandGFP10x17.avi: 18.86s	47,80		CAP49eHandGFP10x18.avi: 30.00s	56,09
	Average	91,15			48,97			56,06
	Standard deviation	6,04			6,31			12,68
	MAX	103,81			57,78			85,38
	MIN	82,35			32,25			44,18

max	CAP49eHandGFP10x20_R3D.avi: 0.86s	103,77	max	CAP49eHandGFP10x21_R3D.avi: 36.71s	51,54
	CAP49eHandGFP10x20_R3D.avi: 1.14s	101,21		CAP49eHandGFP10x21_R3D.avi: 37.14s	53,66
	CAP49eHandGFP10x20_R3D.avi: 1.43s	119,57		CAP49eHandGFP10x21_R3D.avi: 37.86s	45,44
	CAP49eHandGFP10x20_R3D.avi: 1.86s	91,53		CAP49eHandGFP10x21_R3D.avi: 40.14s	42,46
	CAP49eHandGFP10x20_R3D.avi: 2.14s	92,36		CAP49eHandGFP10x21_R3D.avi: 41.57s	43,42
	CAP49eHandGFP10x20_R3D.avi: 5.00s	104,24		CAP49eHandGFP10x21_R3D.avi: 42.71s	44,60
	CAP49eHandGFP10x20_R3D.avi: 6.00s	120,11		CAP49eHandGFP10x21_R3D.avi: 43.86s	43,32
	CAP49eHandGFP10x20_R3D.avi: 6.56s	122,04		CAP49eHandGFP10x21_R3D.avi: 44.86s	47,54
	CAP49eHandGFP10x20_R3D.avi: 7.57s	116,85		CAP49eHandGFP10x21_R3D.avi: 46.14s	42,06
	CAP49eHandGFP10x20_R3D.avi: 7.86s	107,99		CAP49eHandGFP10x21_R3D.avi: 47.00s	46,97
		107,97			46,10
		10,72			3,70
		122,04			53,66
		91,53			42,06
min	CAP49eHandGFP10x20_R3D.avi: 1.00s	74,32	min	CAP49eHandGFP10x21_R3D.avi: 36.29s	35,15
	CAP49eHandGFP10x20_R3D.avi: 1,29s	77,18		CAP49eHandGFP10x21_R3D.avi: 37.00	39,21
	CAP49eHandGFP10x20_R3D.avi: 1,57s	83,19		CAP49eHandGFP10x21_R3D.avi: 37.57	39,53
	CAP49eHandGFP10x20_R3D.avi: 2.00s	89,69		CAP49eHandGFP10x21_R3D.avi: 39.14	35,51
	CAP49eHandGFP10x20_R3D.avi: 2.29s	88,14		CAP49eHandGFP10x21_R3D.avi:40.71s	36,98
	CAP49eHandGFP10x20_R3D.avi: 5.14s	78,26		CAP49eHandGFP10x21_R3D.avi: 42.00	39,43
	CAP49eHandGFP10x20_R3D.avi: 6.14s	69,32		CAP49eHandGFP10x21_R3D.avi: 43.29	38,02
	CAP49eHandGFP10x20_R3D.avi: 7.00s	90,35		CAP49eHandGFP10x21_R3D.avi: 44.43	35,61
	CAP49eHandGFP10x20_R3D.avi: 7.43s	78,75		CAP49eHandGFP10x21_R3D.avi: 45.29	37,39
	CAP49eHandGFP10x20_R3D.avi: 7.71s	69,77		CAP49eHandGFP10x21_R3D.avi: 46.43	34,04
		79,90			37,09
		7,35			1,86
		90,35			39,53
		69,32			34,04

2.3. Evaluation of heart contraction data

Evaluation life imaging heartbeat AFS: Apparent Fractional Shortening [%]			
HandGFP; CAP49e.	HandGFP;CAP49e	HandGFP;w1118	
Sample No.	AFS [%]	Sample No.	AFS[%]
2	17,12	1	54,56
3	30,05	2	52,07
4	2,36	3	39,19
5	0,31	AVERAGE	48,61
7	41,17	STABW.S	8,25
8	31,55	MAX	54,56
9	9,66	MIN	39,19
12	0,58		
13	54,35		
15	3,00		
16	22,09		
17	51,41		
18	1,60		
20	26,00		
21	19,50		
Average	20,72		
Standard deviation	18,32		
MAX	54,35		
MIN	0,31		

Genotype	HandGFP; w1118	HandGFP; CAP49e
sample size	3,00	15,00
Larva #1	54,56	17,12
Larva #2	52,07	30,05
Larva #3	39,19	2,36
Larva #4		0,31
Larva #5		41,17
Larva #6		31,55
Larva #7		9,66
Larva #8		0,58
Larva #9		54,35
Larva #10		3,00
Larva #11		22,09
Larva #12		51,41
Larva #13		1,60
Larva #14		26,00
Larva #15		19,50
Average	48,61	20,72
Stand. Deviation	8,25	18,32
Variance	68,06	335,77
t.test (type3) p score (to w1118)	1	0,004366096
significance (to wild type) p<0.05=*; p<0.01=**	n.s.	**

3. **Evaluation of heart transport activity by dye angiography**

3.1 Transport rate as average pixel intensity in ROI (w^{1118})

sec	w1118-1	w1118-2	w1118-3	Average w1118 1-3	Stand. Dev. w1118 1-3
0	10,51	4,38	25,48	13,46	8,86
20	3,26	2,25	0,42	1,98	1,18
40	13,72	15,60	0,00	9,77	6,95
60	46,42	24,96	4,84	25,41	16,98
80	44,51	53,73	10,41	36,21	18,63
100	49,40	66,57	40,89	52,29	10,68
120	50,60	71,66	44,56	55,60	11,62
140	47,42	77,47	49,76	58,21	13,65
160	49,65	73,85	56,54	60,01	10,18
180	47,18	77,84	58,89	61,30	12,63

3.2 Transport rate as average pixel intensity in ROI (CAP^{42b})

sec	CAP42b-1	CAP42b-2	CAP42b-3	CAP42b-4	CAP42b-5	CAP42b-6	CAP42b-7	Average CAP42b1-7	Stand. Dev. CAP42b1-7
0	0,496	8,341	6,638	6,857	0,000	7,305	11,717	5,908	4,228
20	3,960	1,047	0,000	7,993	4,766	0,000	0,400	2,595	3,066
40	3,847	0,027	0,499	0,384	4,713	5,059	0,000	2,076	2,340
60	4,324	0,193	1,273	0,188	7,024	33,287	0,760	6,721	11,987
80	3,793	0,361	4,565	0,134	7,002	46,024	17,431	11,330	16,370
100	3,868	0,213	7,786	0,690	7,666	57,923	24,699	14,692	20,772
120	2,664	0,386	15,345	0,622	23,508	69,581	25,753	19,694	24,445
140	2,660	0,195	15,530	0,612	24,390	69,394	25,596	19,768	24,423
160	2,705	0,060	15,486	0,473	24,857	69,512	25,324	19,774	24,500
180	2,800	0,000	15,221	0,328	25,170	69,467	25,300	19,755	24,519

3.3 Transport rate as average pixel intensity in ROI (CAP^{49e})

sec	CAP49e-1	CAP49e-2	CAP49e-3	CAP49e-4	CAP49e-5	CAP49e-6	Average CAP49e 1-6	Stand. Dev. CAP49e 1-6
0	17,48	9,36	8,46	7,07	3,40	0,82	7,76	5,75
20	14,84	0,66	8,22	1,12	0,68	0,00	4,25	6,02
40	14,73	0,40	11,87	3,66	13,90	5,41	8,33	5,96
60	14,58	0,10	0,00	3,80	15,87	3,47	6,30	7,11
80	0,00	0,31	2,99	6,07	15,71	13,48	6,43	6,73
100	3,64	0,47	2,67	7,28	15,22	21,76	8,51	8,30
120	4,76	0,72	4,11	9,44	14,55	23,07	9,44	8,23
140	5,83	0,49	3,97	9,39	14,57	39,46	12,28	14,16
160	6,65	1,42	6,73	8,63	13,80	42,49	13,28	14,85
180	5,35	1,37	10,53	7,89	38,81	48,94	18,81	19,90

3.4 Evaluation of transport rate data

	sec	w1118 control	CAP42b	CAP49e
	20	1,98	2,60	4,25
	40	9,77	2,08	8,33
	60	25,41	6,72	6,30
	80	36,21	11,33	6,43
	100	52,29	14,69	8,51
	120	55,60	19,69	9,44
	140	58,21	19,77	12,28
	160	60,01	19,77	13,28
	180	61,30	19,76	18,81
t.test (2;3)		1	0,00717222	0,0038543
significance (to w1118		n.s.	**	**
